# Supplementary material for: Bipyridine Ruthenium(II) Complexes with Halogen-Substituted Salicylates: Synthesis, Crystal Structure, and Biological Activity
Source: Molecules. 2023 Jun 7;28(12):4609. doi: 10.3390/molecules28124609 (PMC10303062; doi:10.3390/molecules28124609)
Supplement: Supplementary file 1 [file molecules-28-04609-s001.zip › molecules-2423244-supplementary.pdf]

Supplementary material

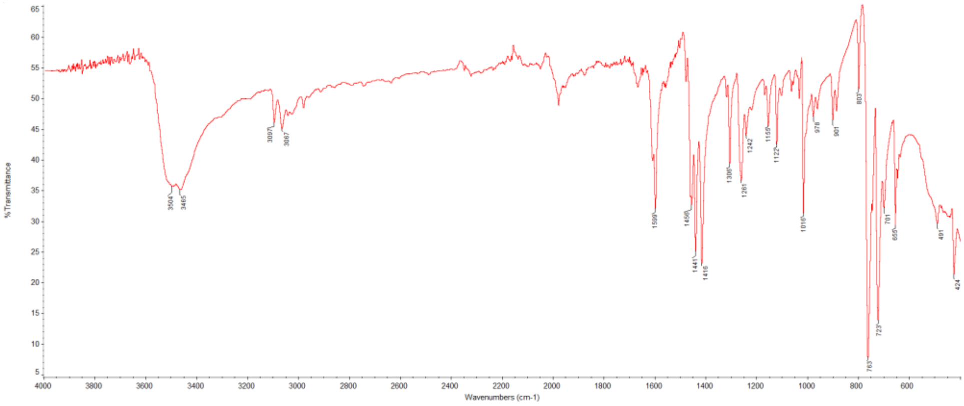

Figure S1 FTIR spectrum of precursor complex [Ru(bipy)$_2$Cl$_2$]

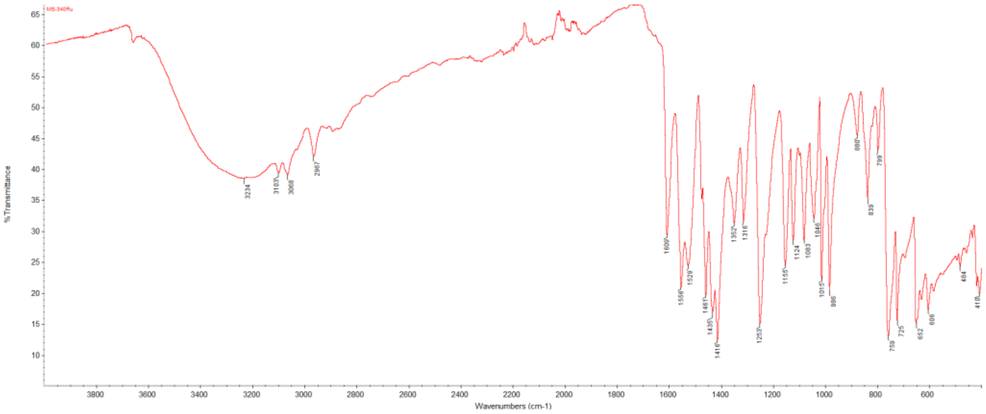

Figure S2 FTIR spectrum of complex [Ru(bipy)$_2$(4-F-Sal)]·3H$_2$O·EtOH (**1**·3H$_2$O·EtOH)

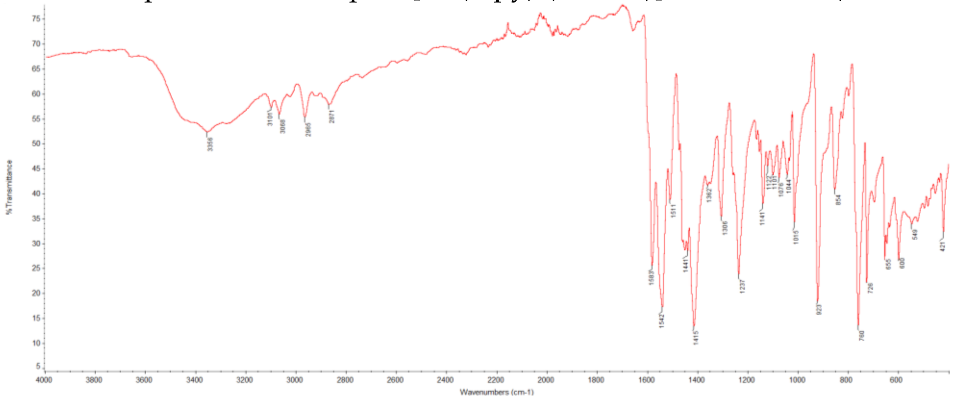

Figure S3 FTIR spectrum of complex [Ru(bipy)$_2$(4-Cl-Sal)]·2.6H$_2$O·2EtOH (**2**·2.6H$_2$O·2EtOH)

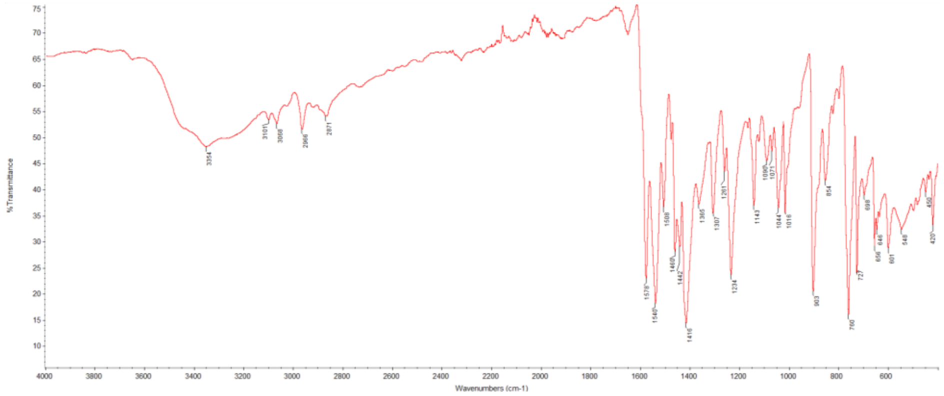

Figure S4 FTIR spectrum of complex [Ru(bipy)$_2$(4-Br-Sal)]·6H$_2$O (**3**·6H$_2$O)

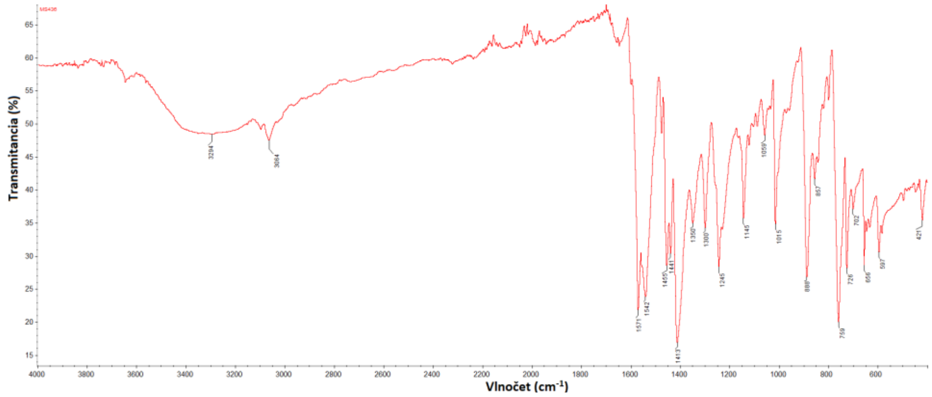

Figure S5 FTIR spectrum of complex [Ru(bipy)$_2$(4-I-Sal)]·3H$_2$O (**4**·3H$_2$O)

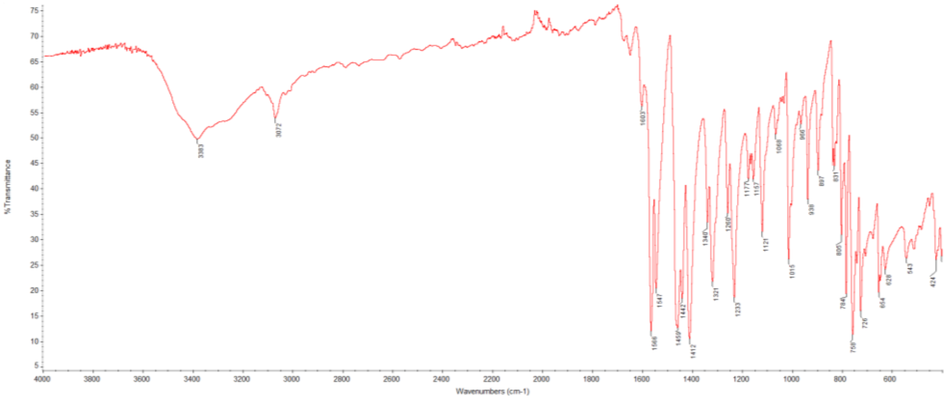

Figure S6 FTIR spectrum of complex [Ru(bipy)$_2$(5-F-Sal)]·1.55H_2O (**5**·1.55H_2O)

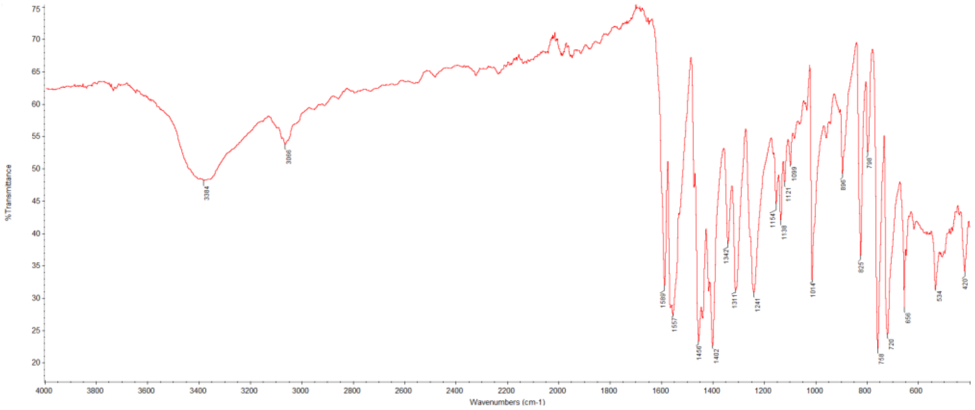

Figure S7 FTIR spectrum of complex [Ru(bipy)$_2$(5-Cl-Sal)] (**6**)

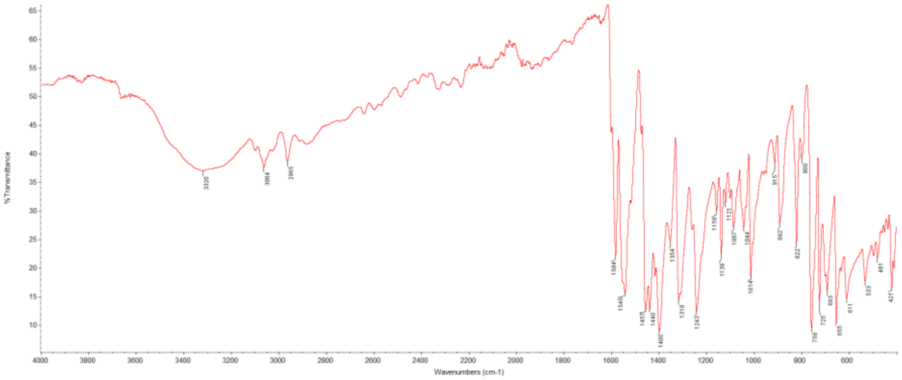

Figure S8 FTIR spectrum of complex [Ru(bipy)$_2$(5-Br-Sal)] (**7**)

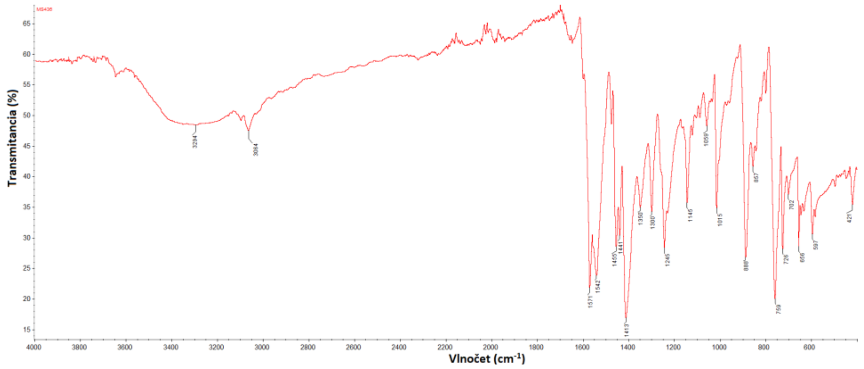

Figure S9 FTIR spectrum of complex [Ru(bipy)$_2$(5-I -Sal)]·4H_2O (**8**·4H_2O)

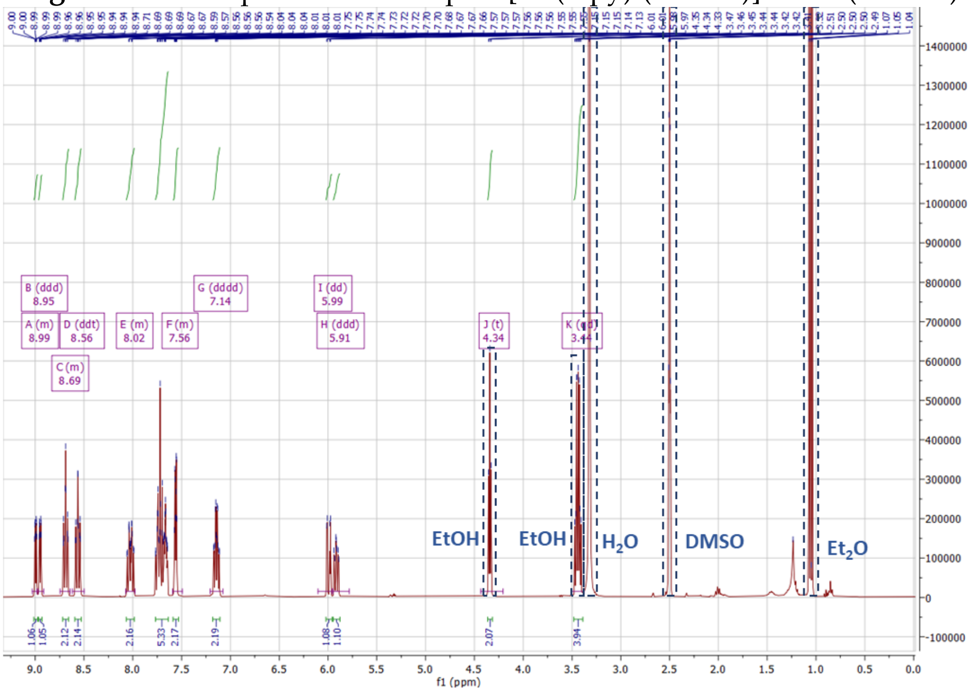

Figure S10 ^{1}H NMR spectrum of complex [Ru(bipy)$_2$(4-F-Sal)] (**1**) with marked signals for solvents and impurities (blue dashed line).

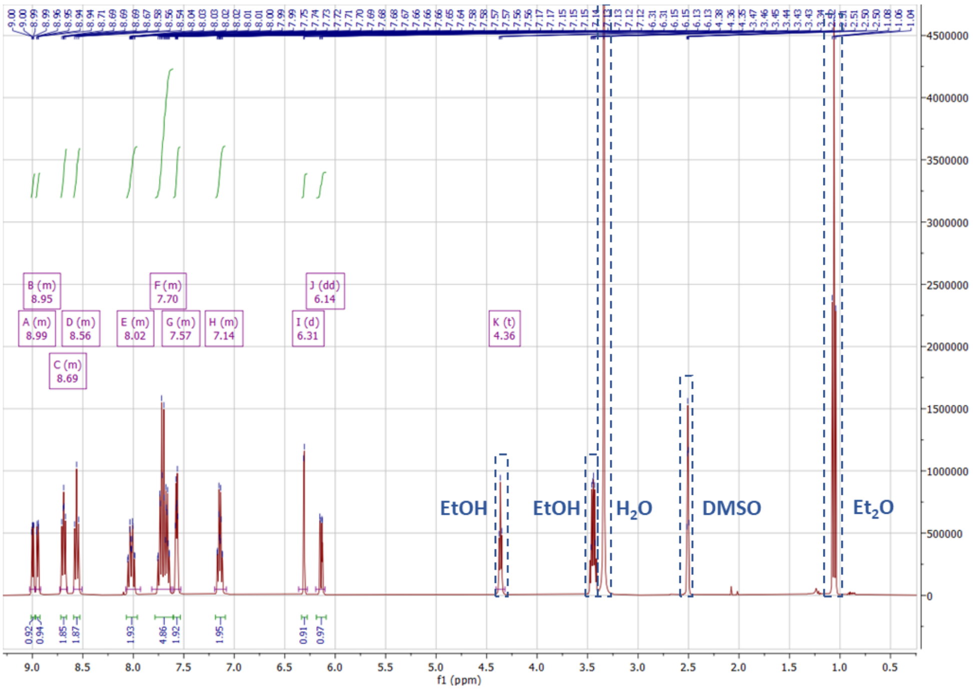

Figure S11 ^{1}H NMR spectrum of complex [Ru(bipy)2(4-Cl-Sal)] (**2**) with marked signals for solvents and impurities (blue dashed line).

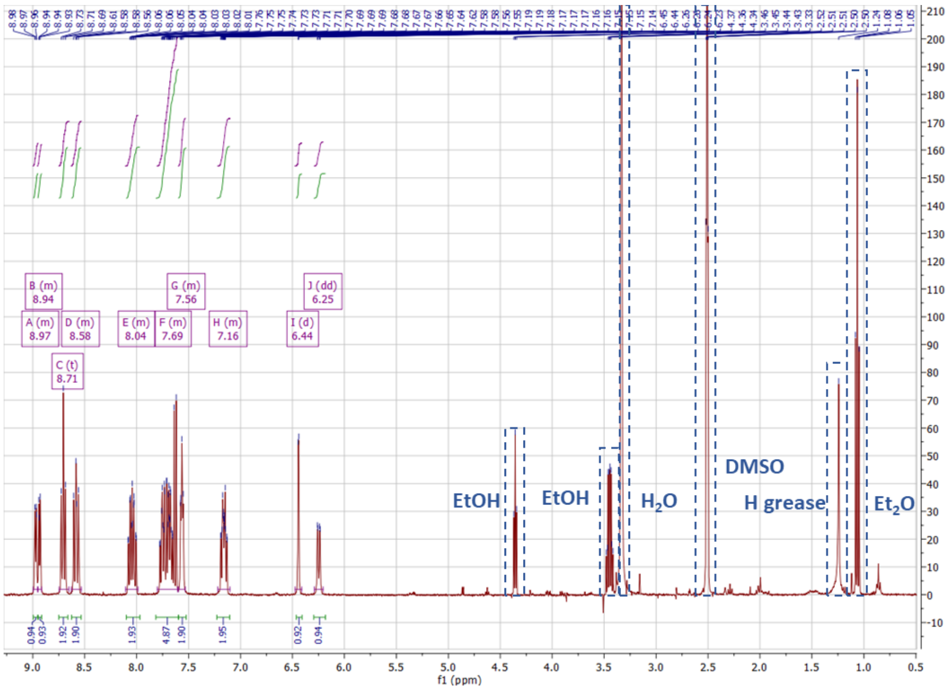

Figure S12 ^{1}H NMR spectrum of complex [Ru(bipy)2(4-Br-Sal)] (**3**) with marked signals for solvents and impurities (blue dashed line).

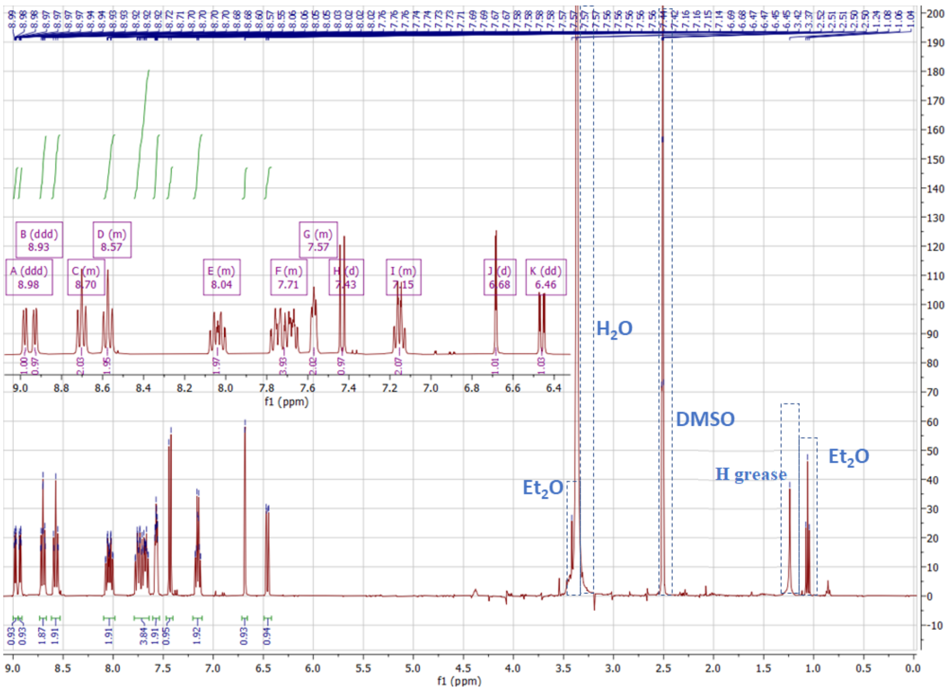

Figure S13 ^{1}H NMR spectrum of complex [Ru(bipy)$_2$(4-I-Sal)] (**4**) with marked signals for solvents and impurities (blue dashed line).

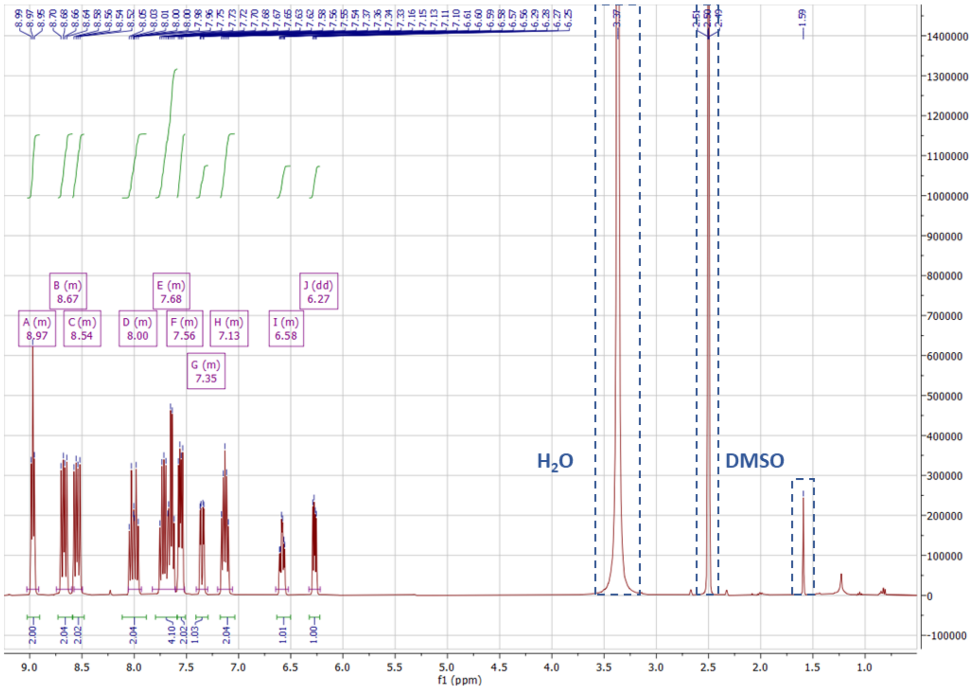

Figure S14 ^{1}H NMR spectrum of complex [Ru(bipy)$_2$(5-F-Sal)] (**5**) with marked signals for solvents and impurities (blue dashed line).

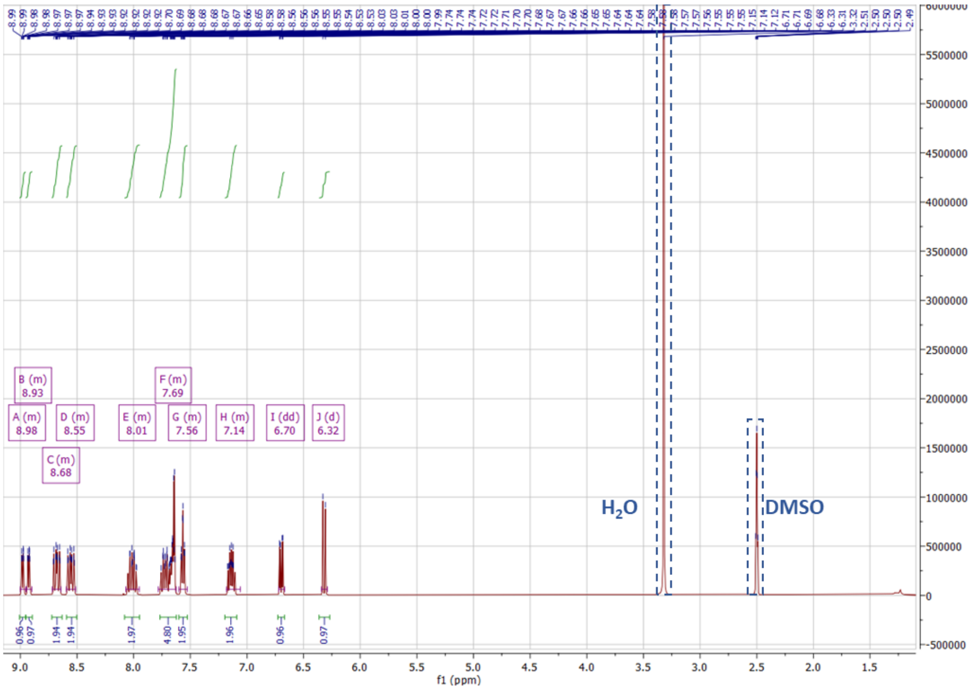

Figure S15 ^{1}H NMR spectrum of complex [Ru(bipy)$_2$(5-Cl-Sal)] (**6**) with marked signals for solvents and impurities (blue dashed line).

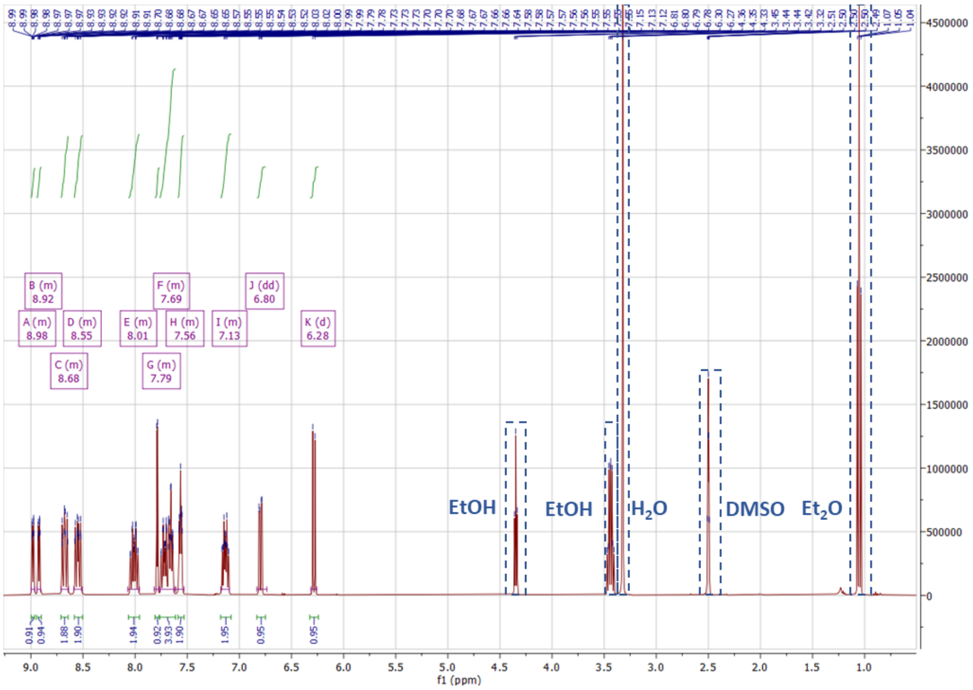

Figure S16 ^{1}H NMR spectrum of complex [Ru(bipy)$_2$(5-Br-Sal)] (**7**) with marked signals for solvents and impurities (blue dashed line).

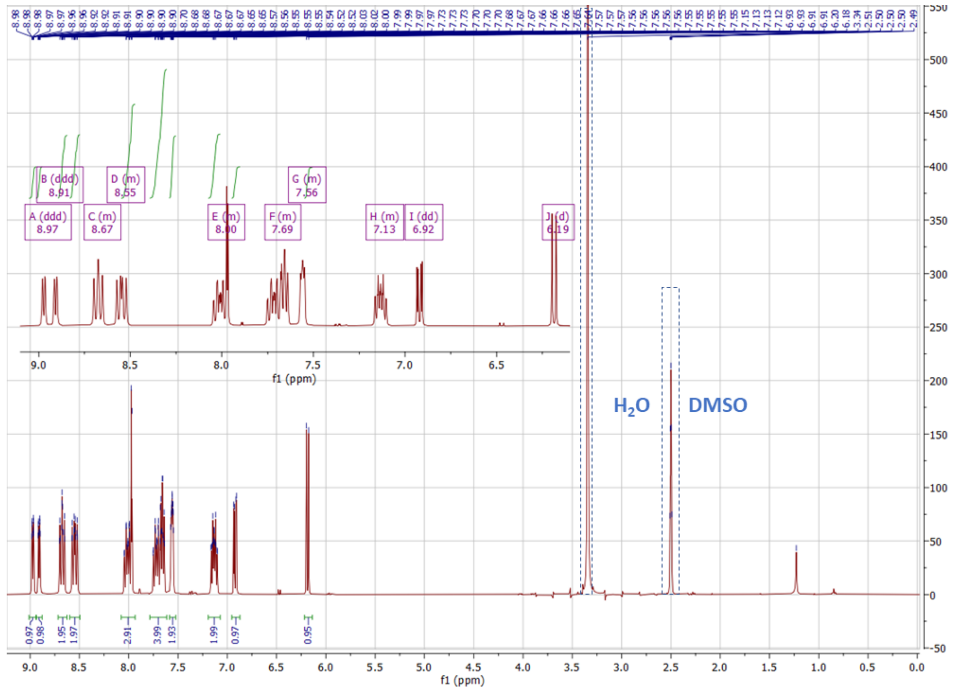

Figure S17 ^{1}H NMR spectrum of complex [Ru(bipy)$_2$(5-I-Sal)] (**8**) with marked signals for solvents and impurities (blue dashed line).

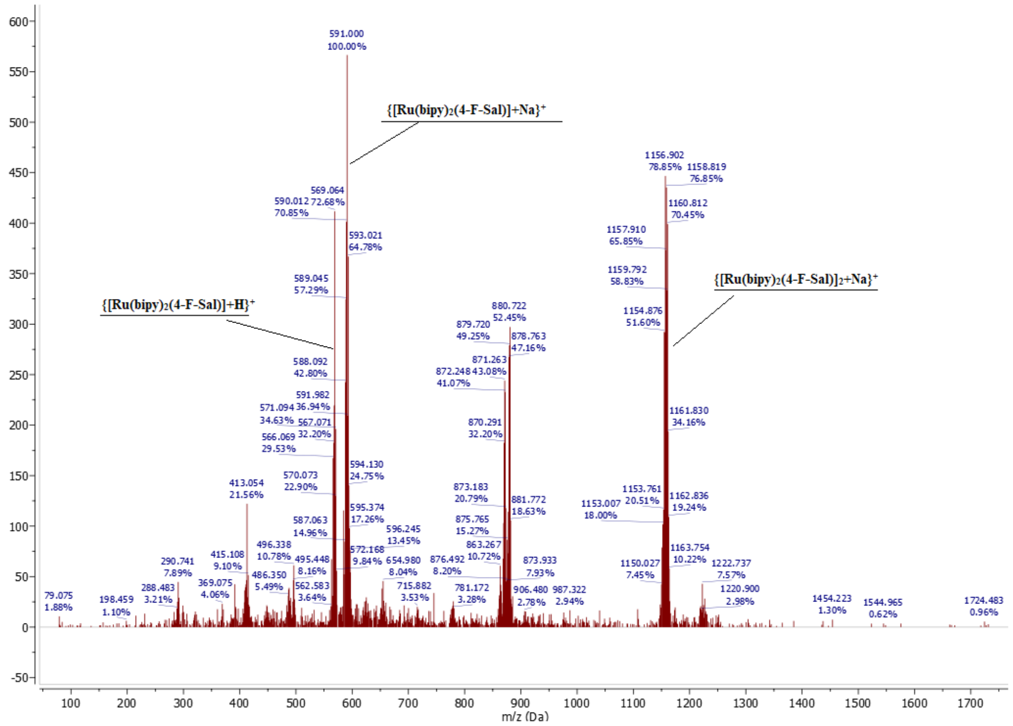

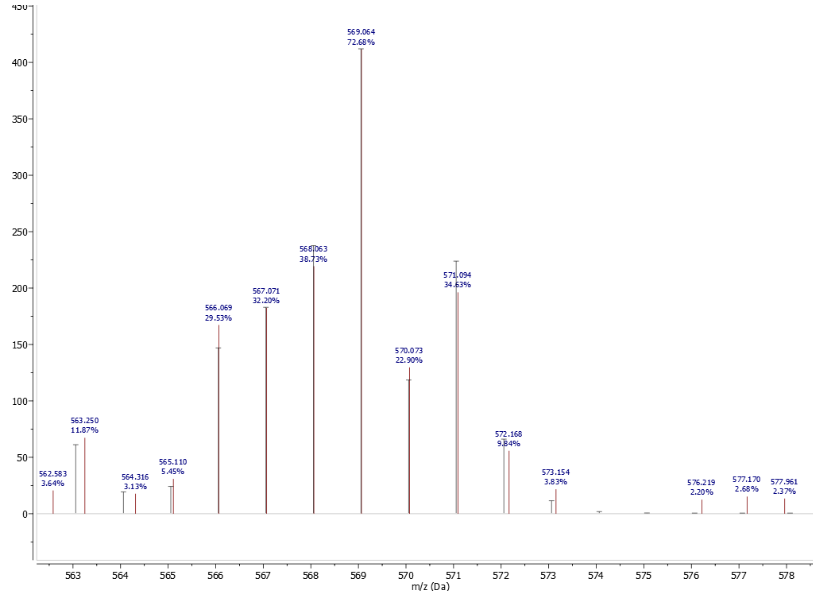

Figure S18 ESI-MS spectrum of complex [Ru(bipy)2(4-F-Sal)] (**1**) together with the assignment of the most significant peaks. Below is a comparison of the simulated and measured isotope pattern for the molecular peak.

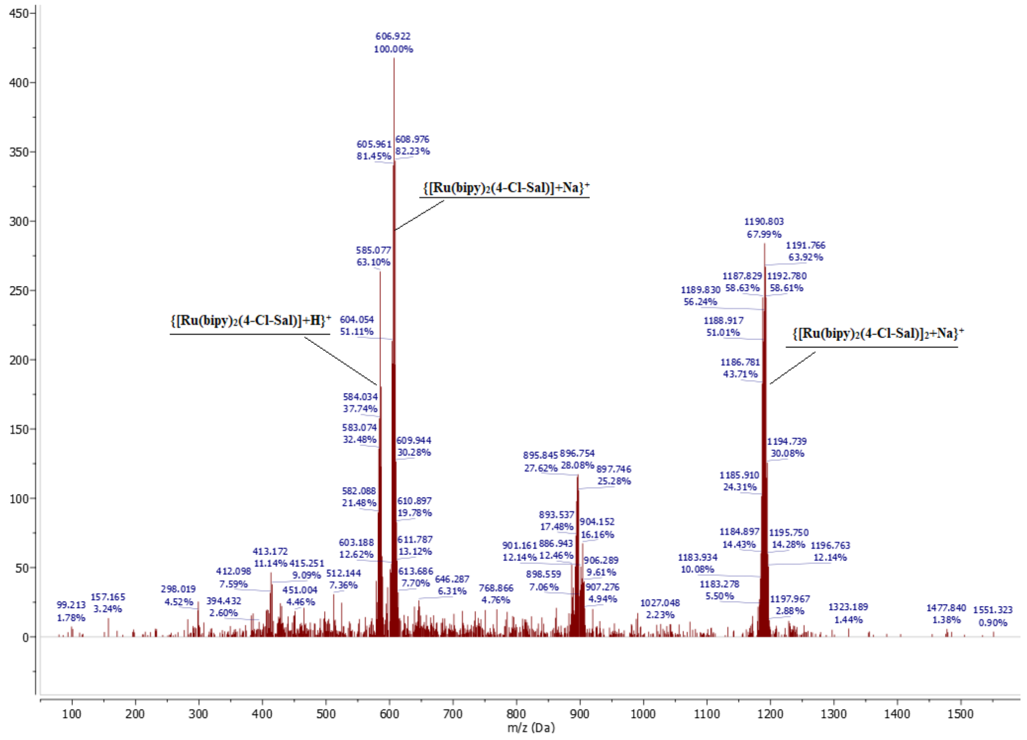

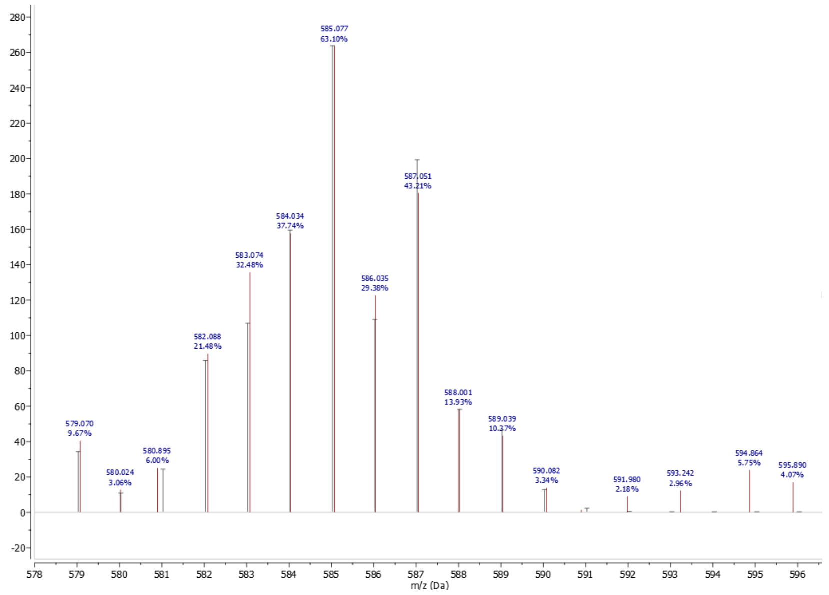

Figure S19 ESI-MS spectrum of complex [Ru(bipy)$_2$(4-Cl-Sal)] (**2**) together with the assignment of the most significant peaks. Below is a comparison of the simulated and measured isotope pattern for the molecular peak.

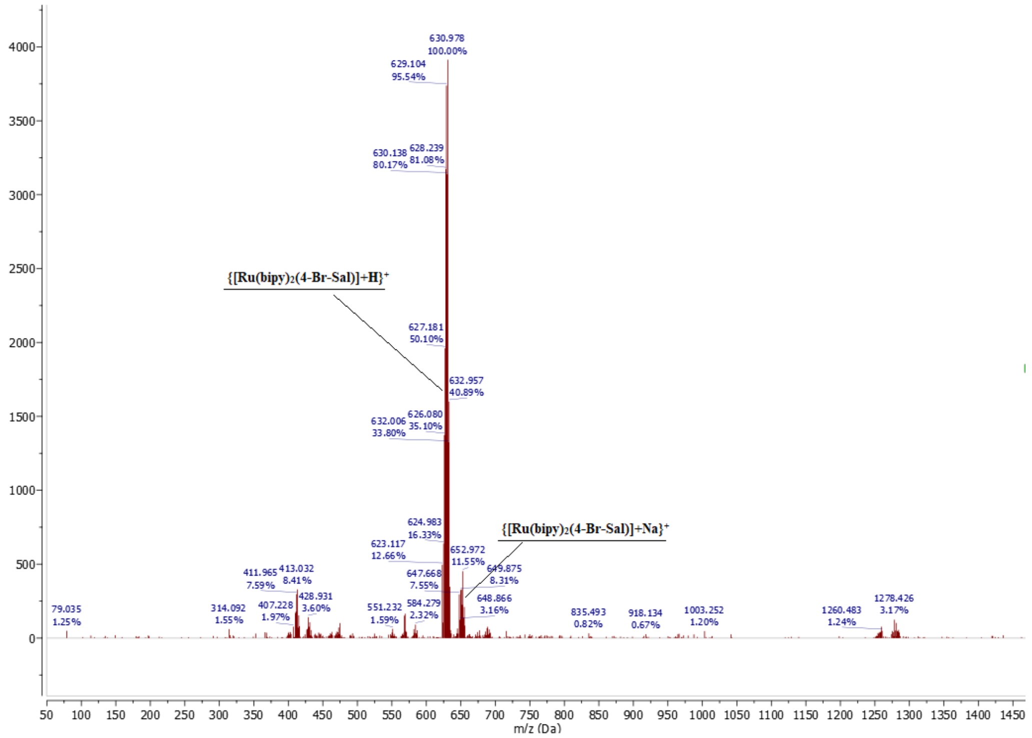

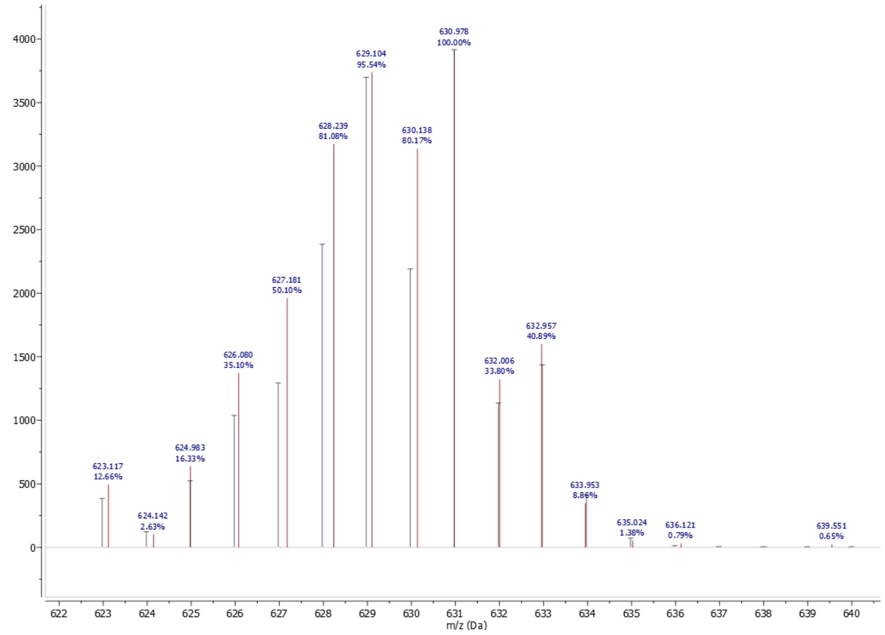

Figure S20 ESI-MS spectrum of complex [Ru(bipy)$_2$(4-Br-Sal)] (**3**) together with the assignment of the most significant peaks. Below is a comparison of the simulated and measured isotope pattern for the molecular peak.

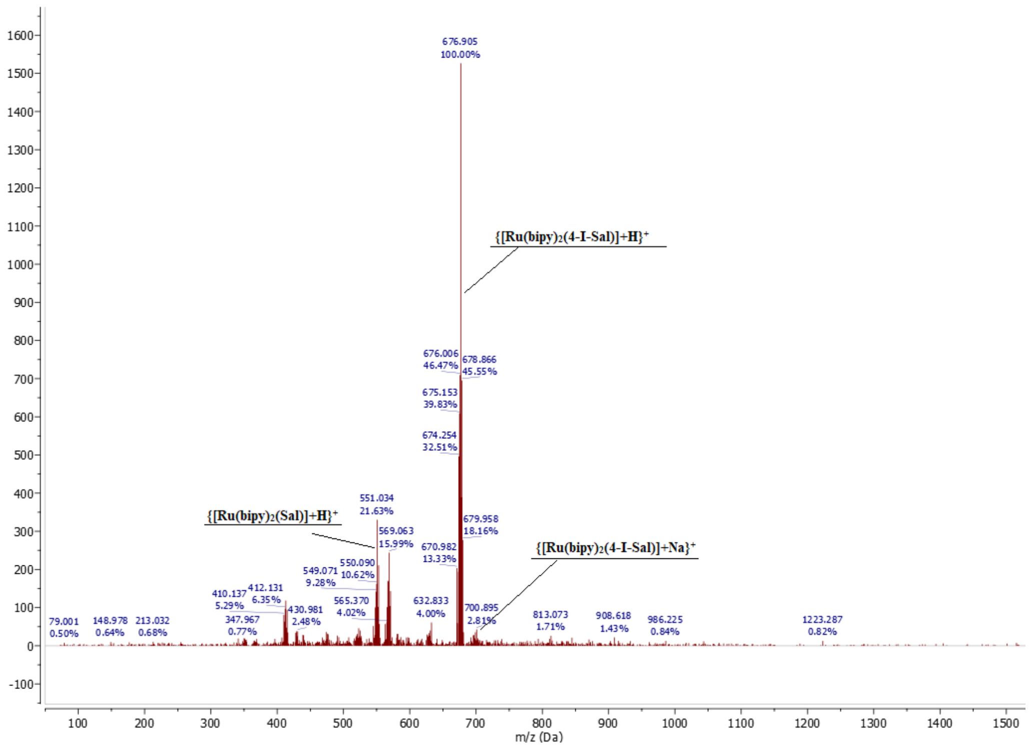

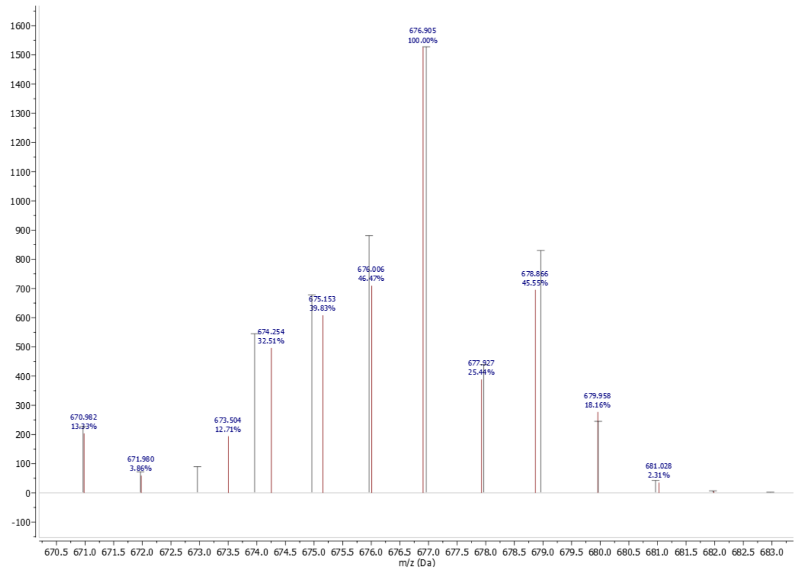

Figure S21 ESI-MS spectrum of complex [Ru(bipy)$_2$(4-I-Sal)] (**4**) together with the assignment of the most significant peaks. Below is a comparison of the simulated and measured isotope pattern for the molecular peak.

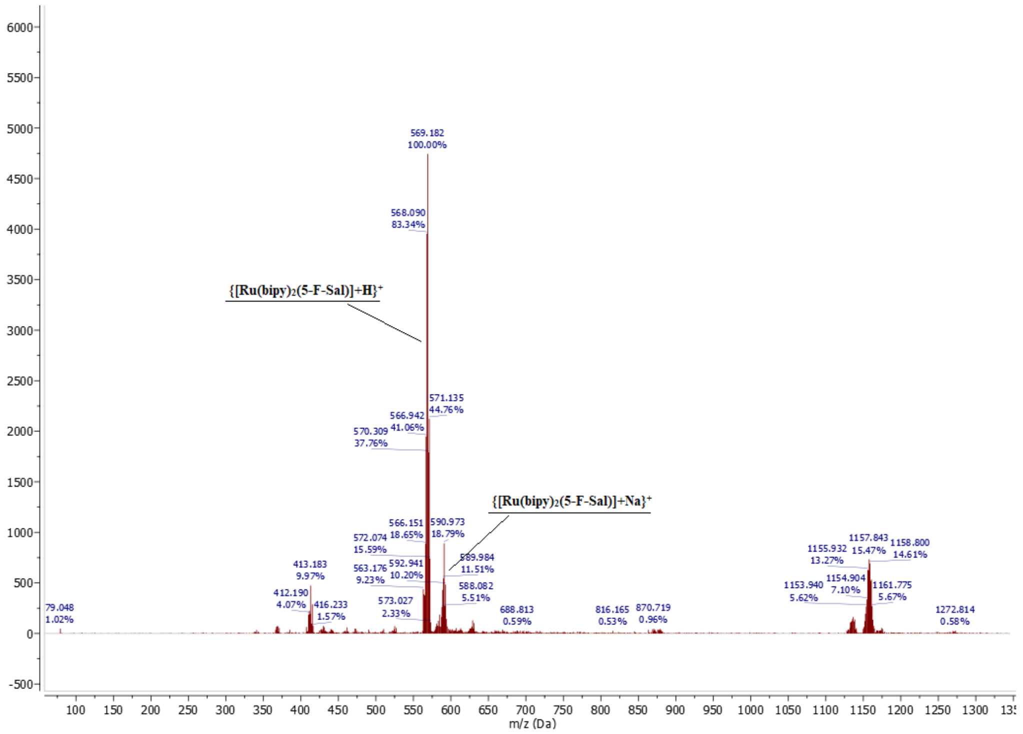

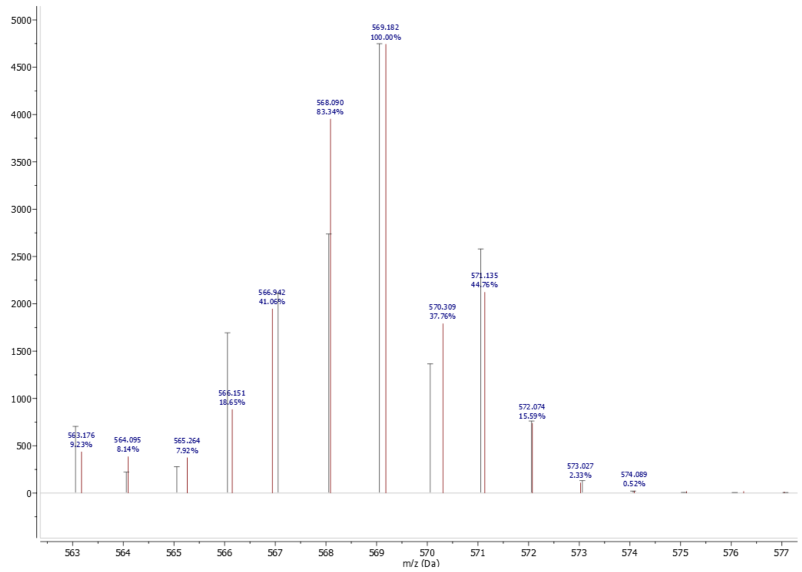

Figure S22 ESI-MS spectrum of complex [Ru(bipy)2(5-F-Sal)] (**5**) together with the assignment of the most significant peaks. Below is a comparison of the simulated and measured isotope pattern for the molecular peak.

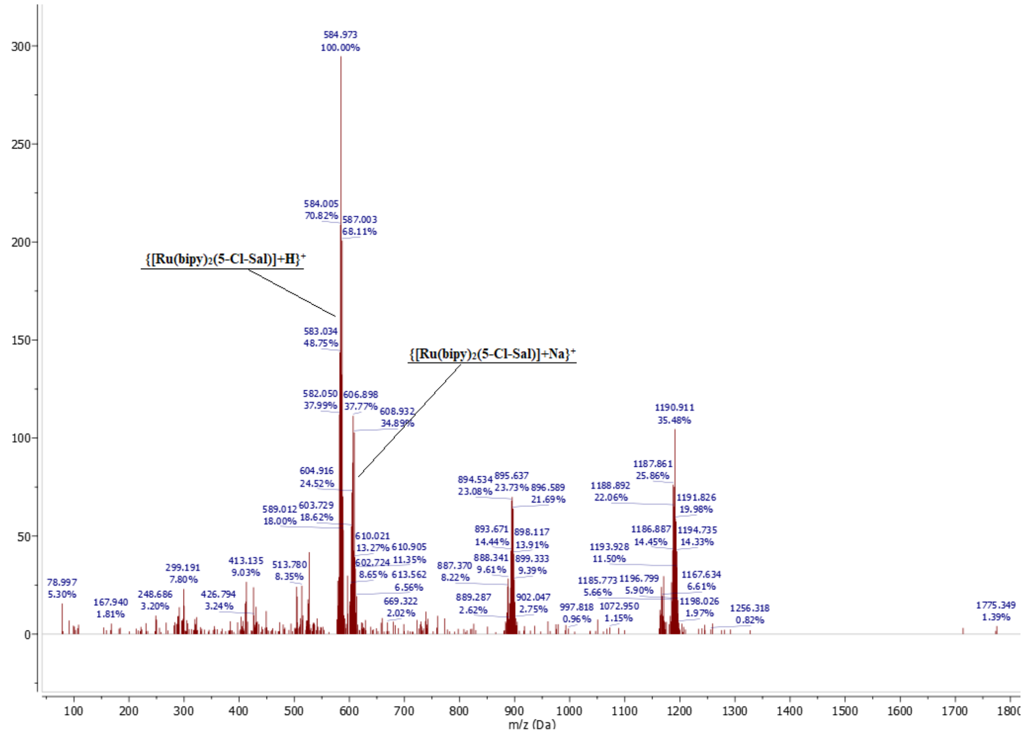

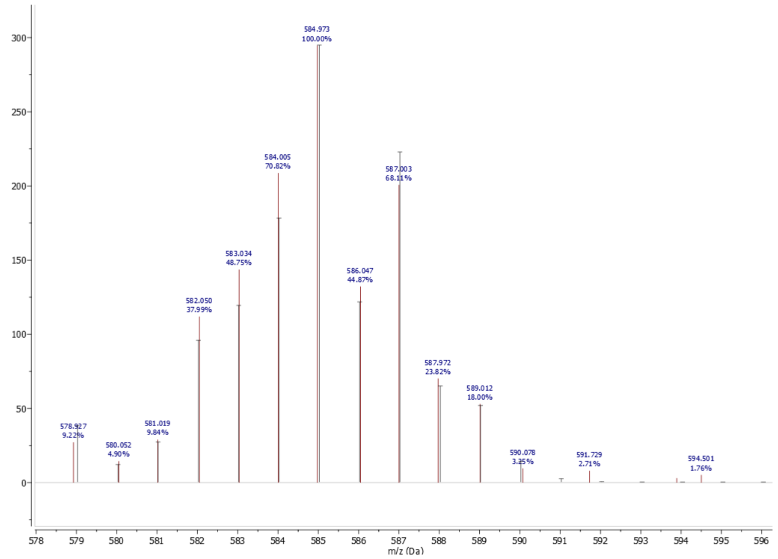

Figure S23 ESI-MS spectrum of complex [Ru(bipy)₂(5-Cl-Sal)] (**6**) together with the assignment of the most significant peaks. Below is a comparison of the simulated and measured isotope pattern for the molecular peak.

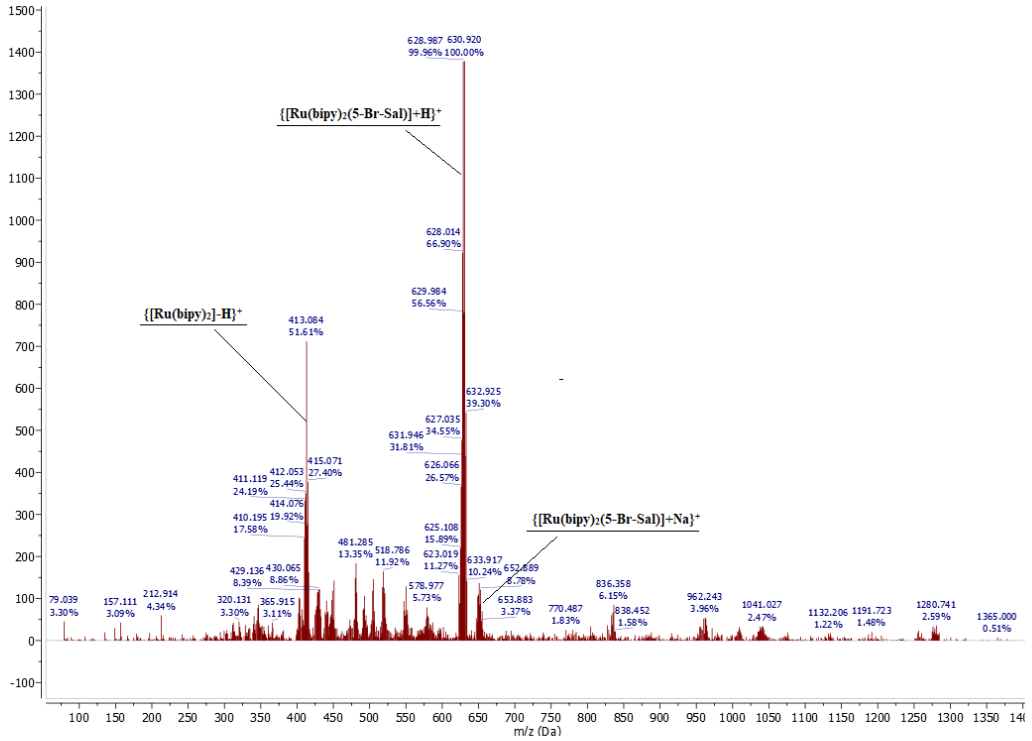

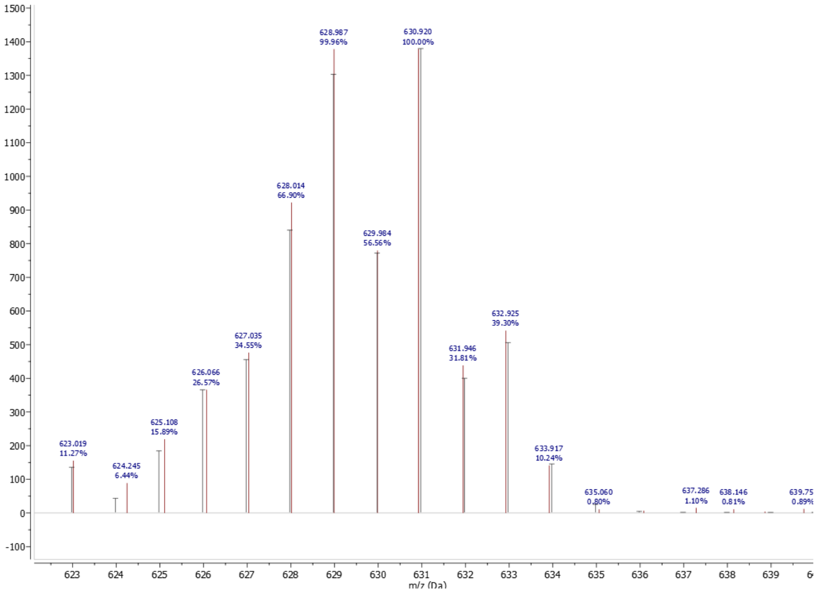

Figure S24 ESI-MS spectrum of complex [Ru(bipy)$_2$(5-Br-Sal)] (**7**) together with the assignment of the most significant peaks. Below is a comparison of the simulated and measured isotope pattern for the molecular peak.

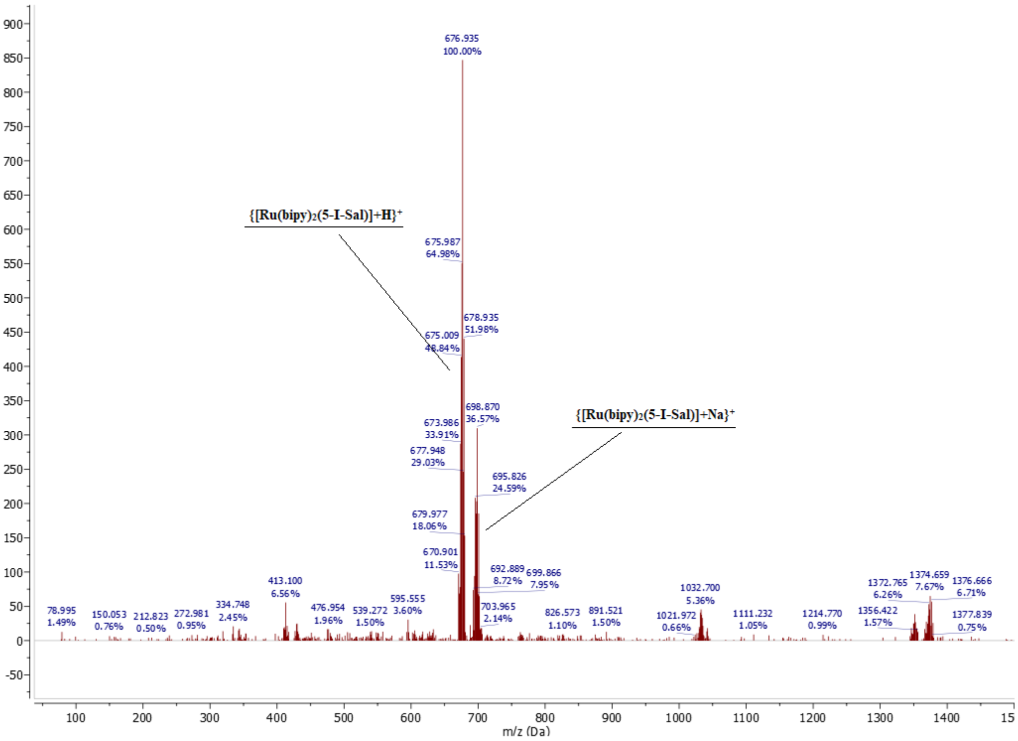

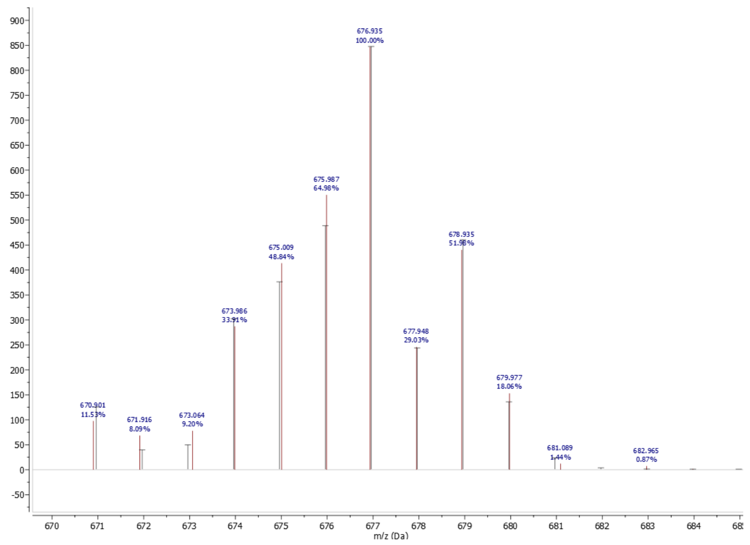

Figure S25 ESI-MS spectrum of complex [Ru(bipy)$_2$(5-I -Sal)] (**8**) together with the assignment of the most significant peaks. Below is a comparison of the simulated and measured isotope pattern for the molecular peak.

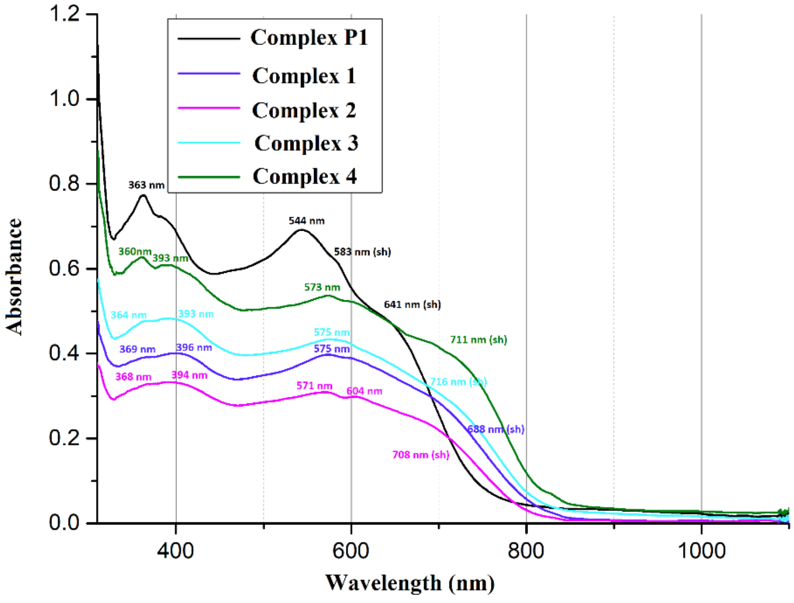

Figure S26 UV-Vis spectra of complexes **1** – **4** measured in solid state as nujol suspension.

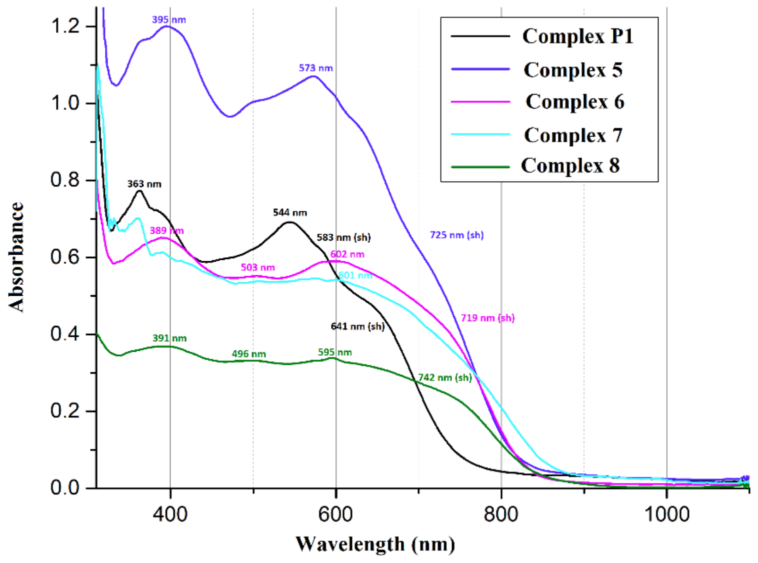

Figure S27 UV-Vis spectra of complexes **5 – 8** measured in solid state as nujol suspension.

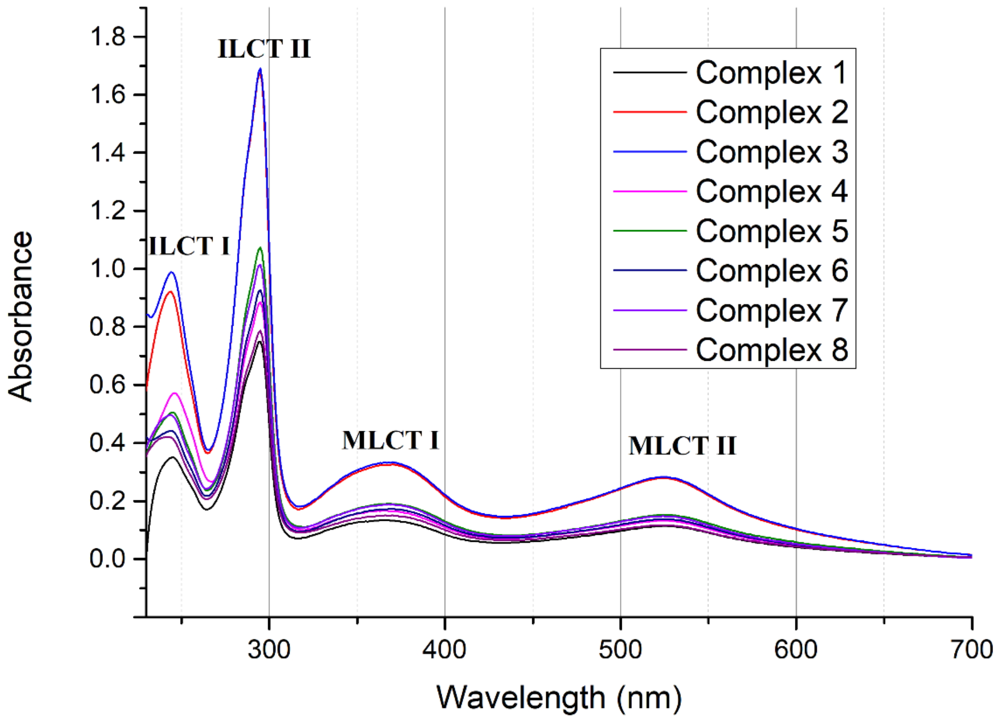

Figure S28 UV-Vis spectra of complexes **1 – 8** measured in solutions.

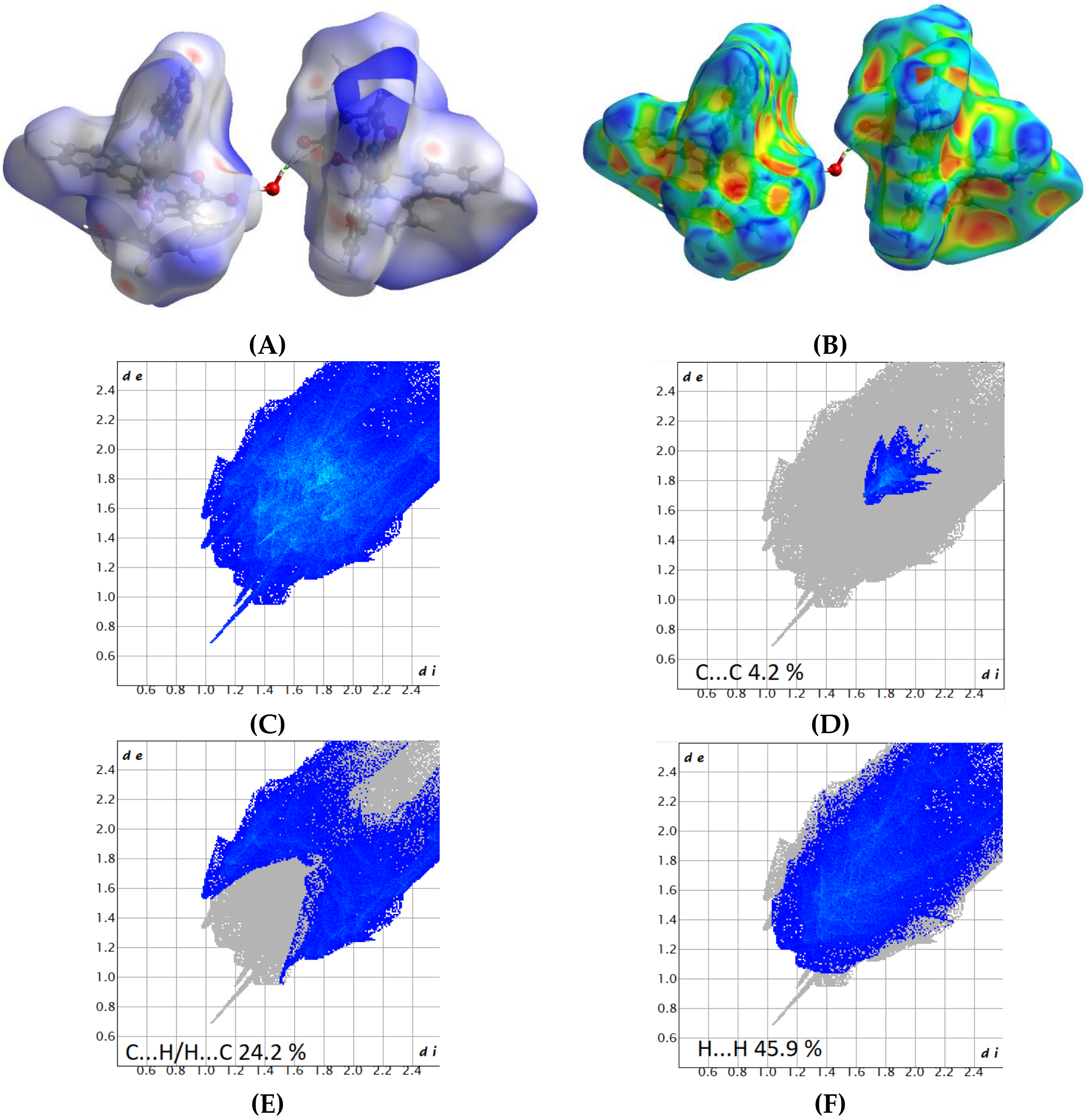

(A) (B)

(C) (D)

(E) (F)

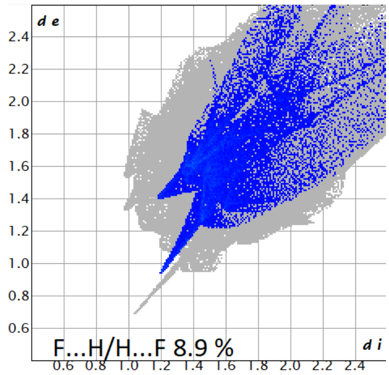

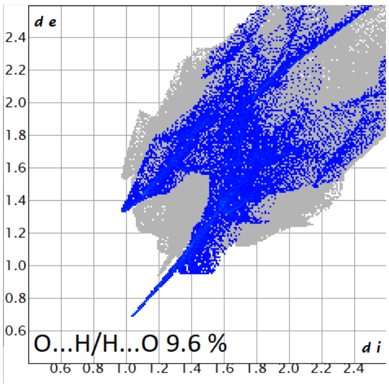

(G) **(H)**

Figure S29 Hirshfeld surface mapped oved d_{norm} (A) and shape index (B) for complex [Ru(bipy)$_2$(4-F-Sal)]·3H_2O·EtOH (**1**·3H_2O·EtOH) together with corresponding overall fingerprint plot (C) and plots by close contacts types: C…C (D); C…H/H…C (E); H…H (F); F…H/H…F (G) and O…H/H…O (H).

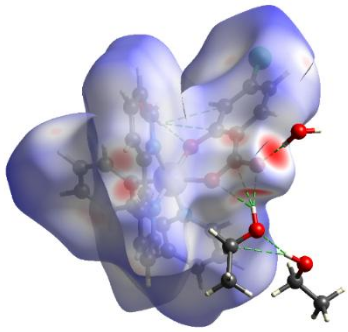

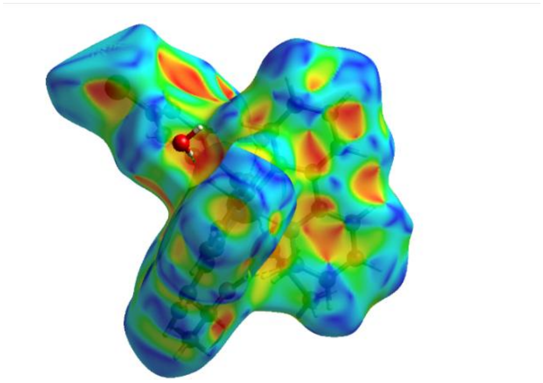

(A) **(B)**

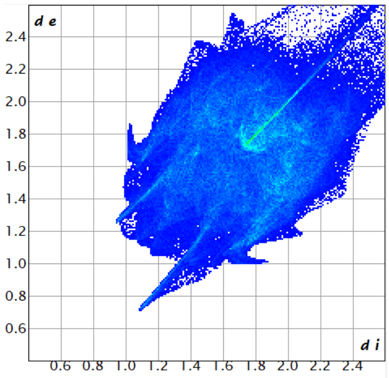

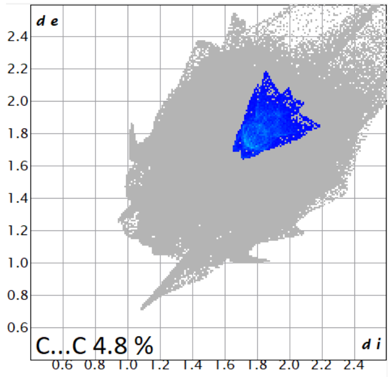

(C) **(D)**

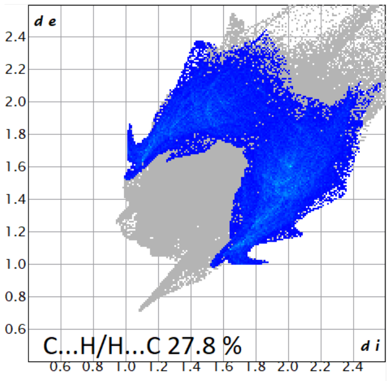

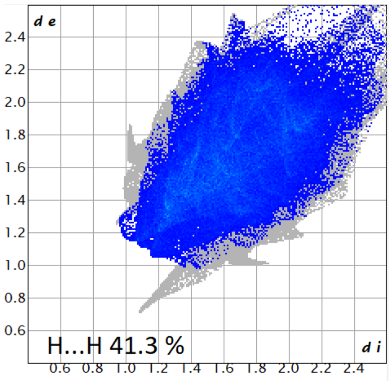

(E) **(F)**

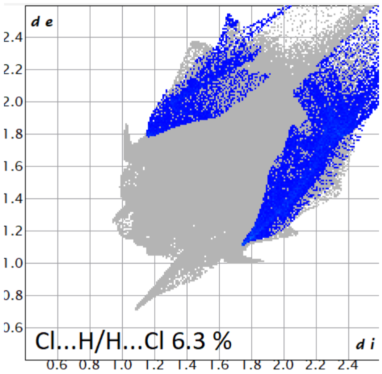

(G)

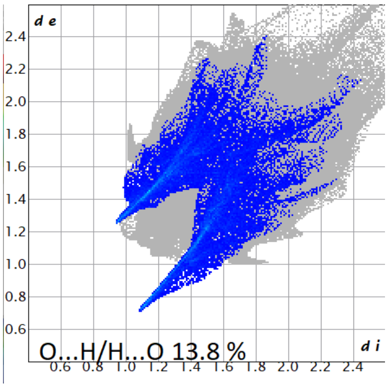

(H)

Figure S30 Hirshfeld surface mapped oved d_{norm} (A) and shape index (B) for complex [Ru(bipy)$_2$(4-Cl-Sal)]·2.6H_2O·2EtOH (**2**·2.6H_2O·2EtOH) together with corresponding overall fingerprint plot (C) and plots by close contacts types: C…C (D); C…H/H…C (E); H…H (F); Cl…H/H…Cl (G) and O…H/H…O (H).

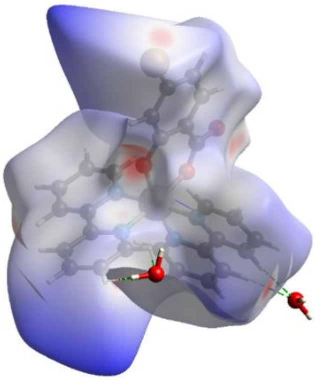

(A)

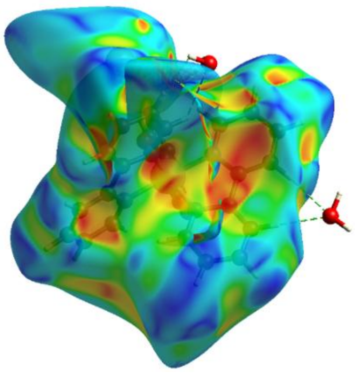

(B)

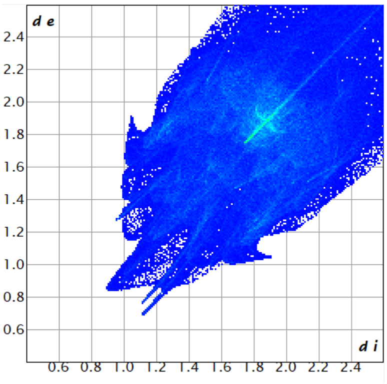

(C)

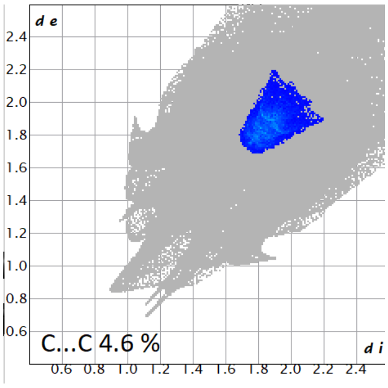

(D)

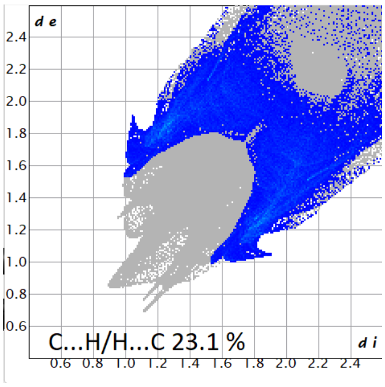

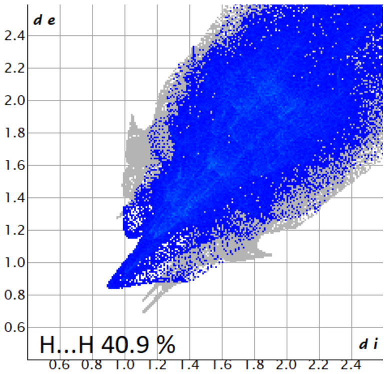

(E) (F)

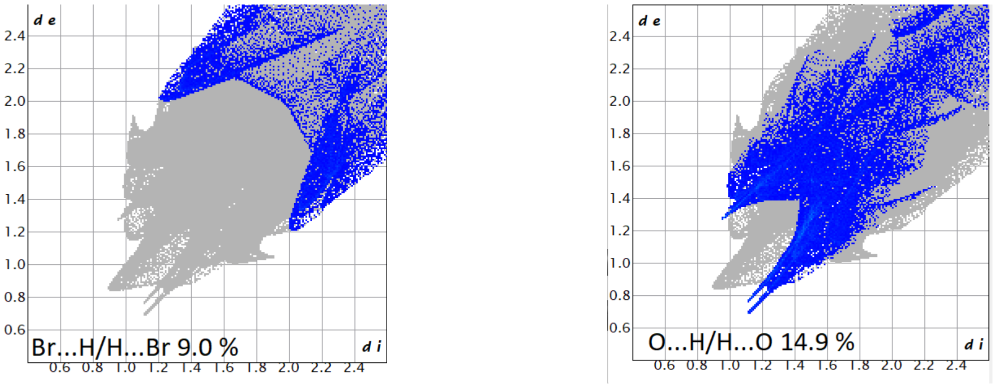

(G) (H)

Figure S31 Hirshfeld surface mapped oved d_{norm} (A) and shape index (B) for complex $[Ru(bipy)_2(4\text{-}Br\text{-}Sal)]\cdot 6H_2O$ (**3**$\cdot 6H_2O$) together with corresponding overall fingerprint plot (C) and plots by close contacts types: C…C (D); C…H/H…C (E); H…H (F); Br…H/H…Br (G) and O…H/H…O (H).

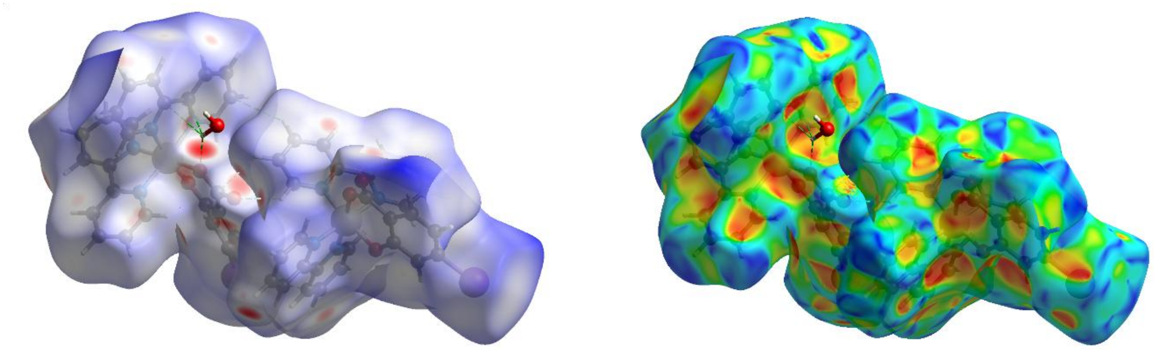

(A) (B)

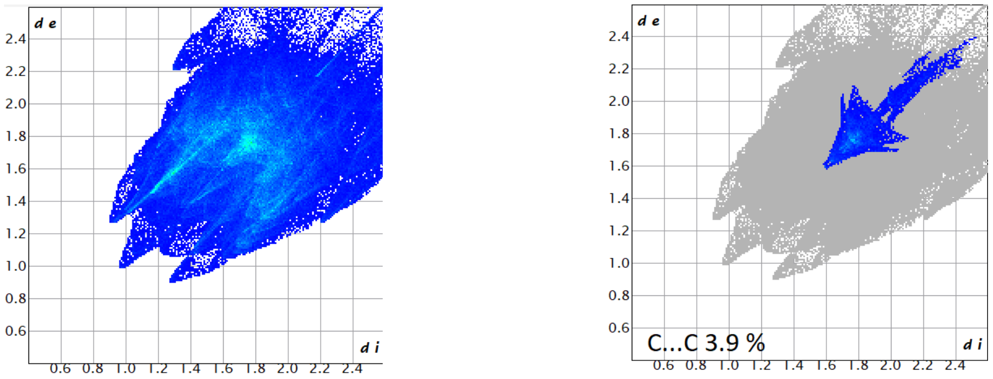

(C) (D)

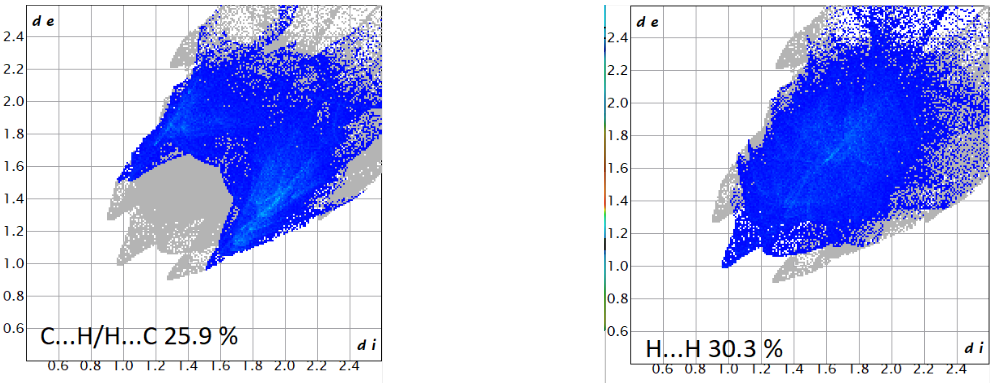

(E)

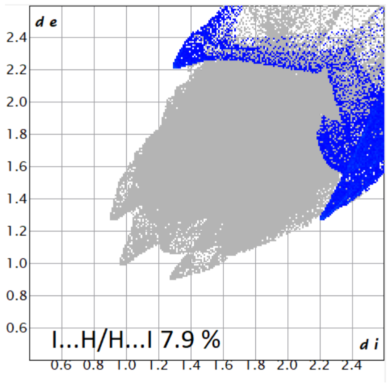

(F)

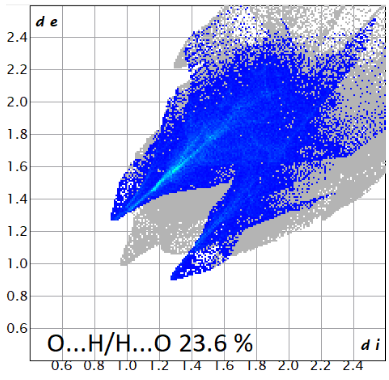

(G) (H)

Figure S32 Hirshfeld surface mapped oved d_{norm} (A) and shape index (B) for complex [Ru(bipy)$_2$(4-I-Sal)]·3H_2O (**4**·3H_2O) together with corresponding overall fingerprint plot (C) and plots by close contacts types: C…C (D); C…H/H…C (E); H…H (F); I…H/H…I (G) and O…H/H…O (H).

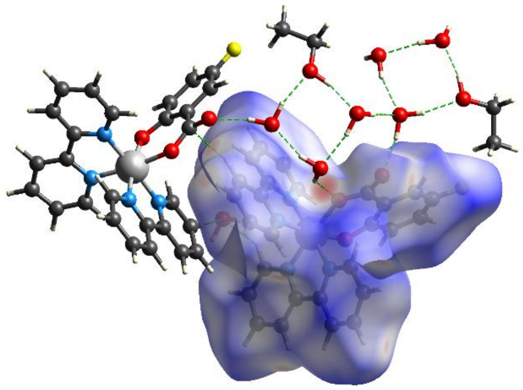

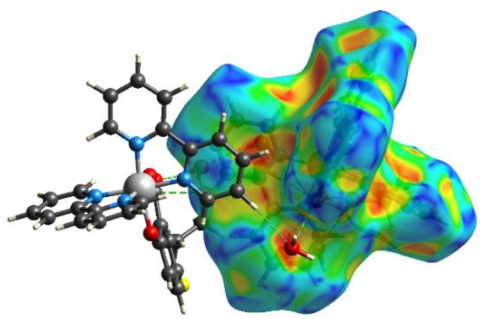

(A) (B)

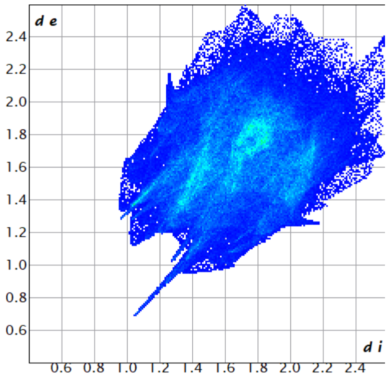

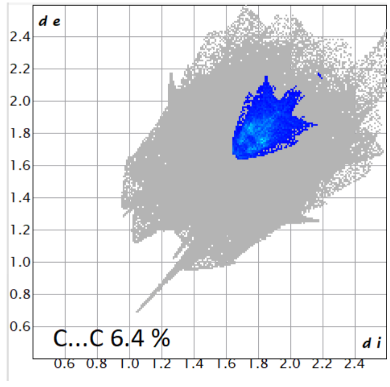

(C) (D)

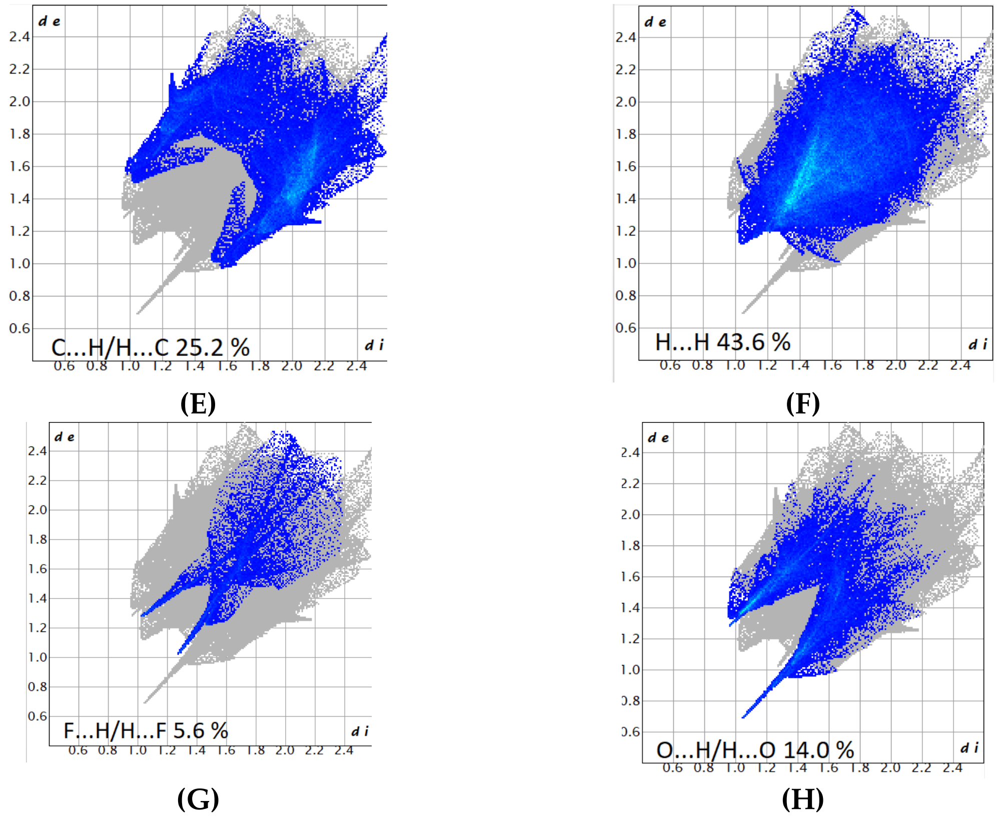

Figure S33 Hirshfeld surface mapped oved d_{norm} (A) and shape index (B) for complex [Ru(bipy)$_2$(5-F-Sal)]·1.55H_2O (**5**·1.55H_2O) together with corresponding overall fingerprint plot (C) and plots by close contacts types: C…C (D); C…H/H…C (E); H…H (F); F…H/H…F (G) and O…H/H…O (H).

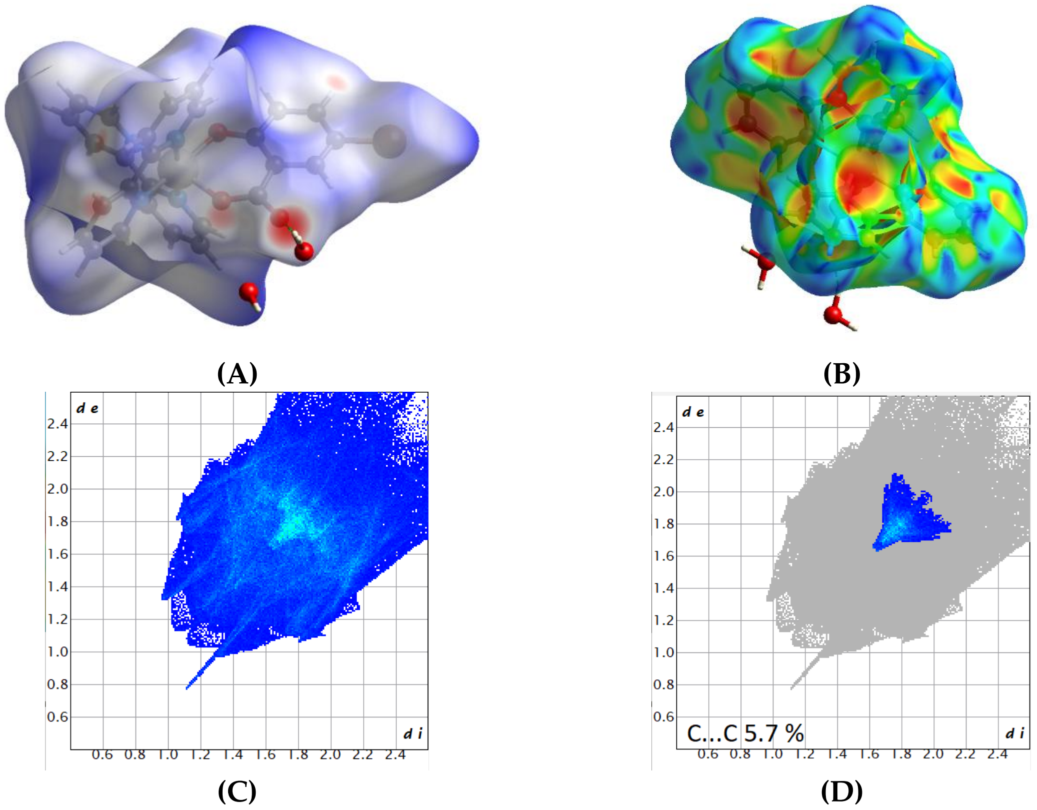

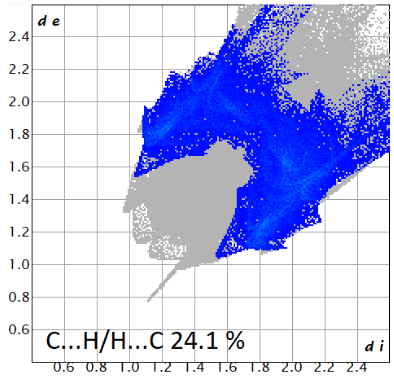

(E)

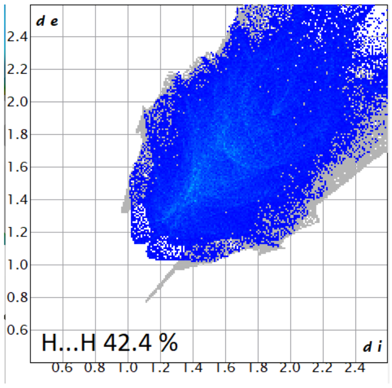

(F)

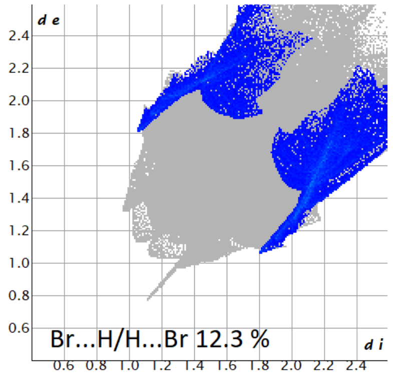

(G)

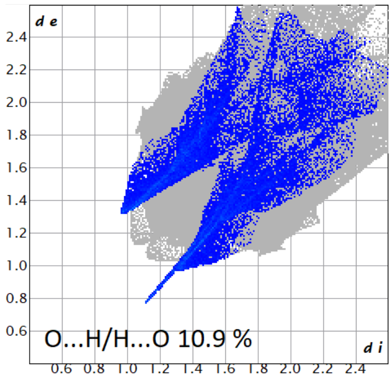

(H)

Figure S34 Hirshfeld surface mapped oved d_{norm} (A) and shape index (B) for complex [Ru(bipy)$_2$(5-Br-Sal)]·1.75H_2O (**7**·1.75H_2O) together with corresponding overall fingerprint plot (C) and plots by close contacts types: C…C (D); C…H/H…C (E); H…H (F); Br…H/H…Br (G) and O…H/H…O (H).

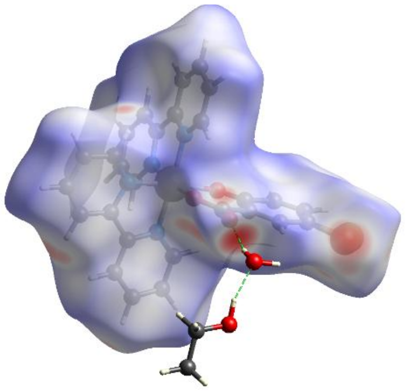

(A)

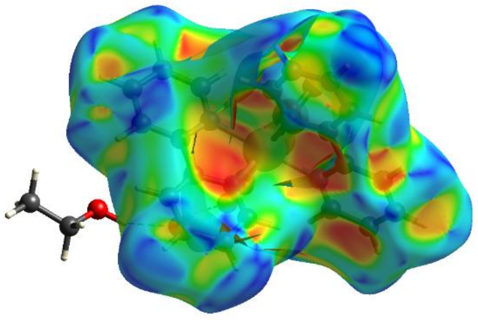

(B)

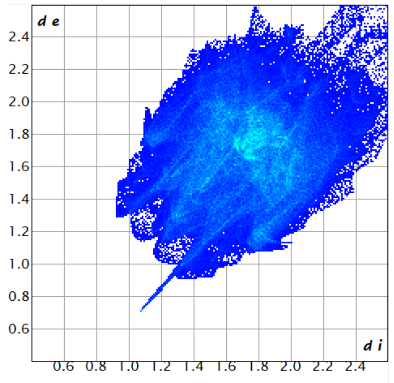

(C)

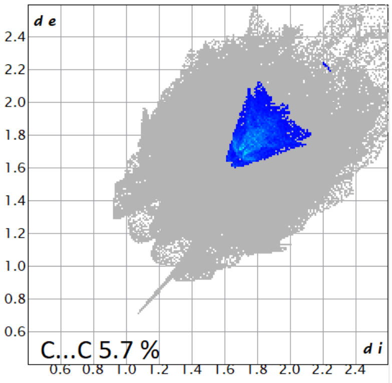

(D)

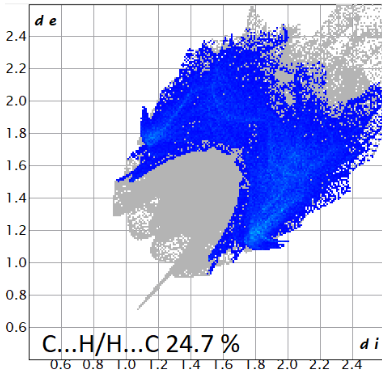

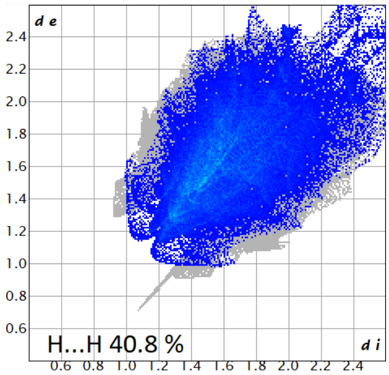

(E) **(F)**

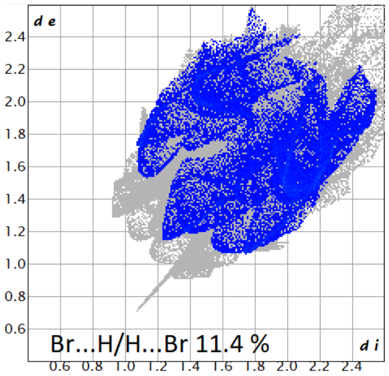

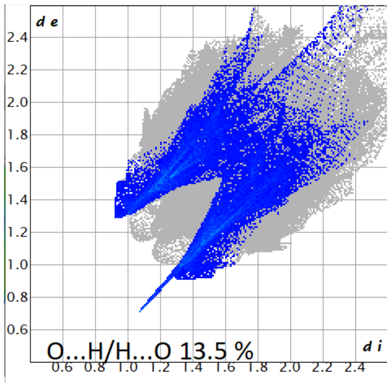

(G) **(H)**

Figure S35 Hirshfeld surface mapped oved d_{norm} (A) and shape index (B) for complex [Ru(bipy)$_2$(5-Br-Sal)]·H_2O·EtOH (**7**·H_2O·EtOH) together with corresponding overall fingerprint plot (C) and plots by close contacts types: C…C (D); C…H/H…C (E); H…H (F); Br…H/H…Br (G) and O…H/H…O (H).

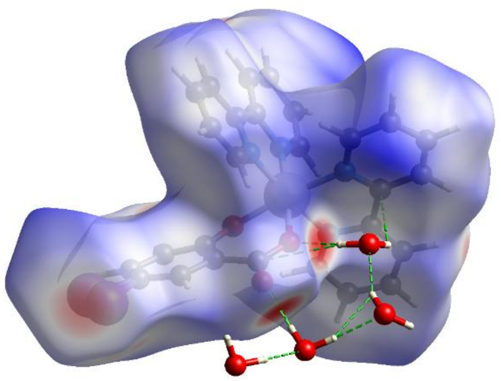

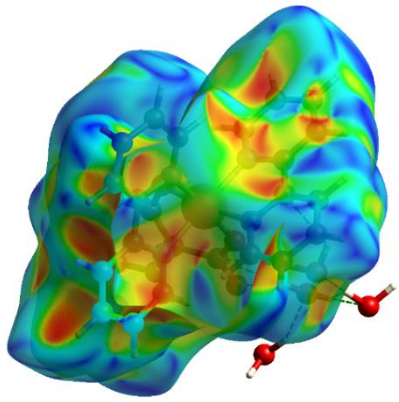

(A) **(B)**

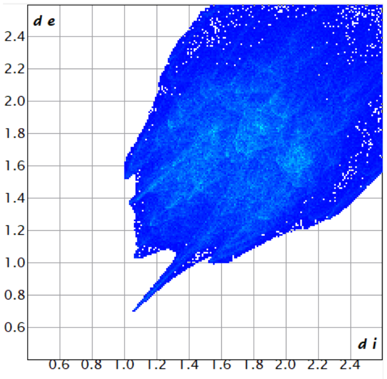

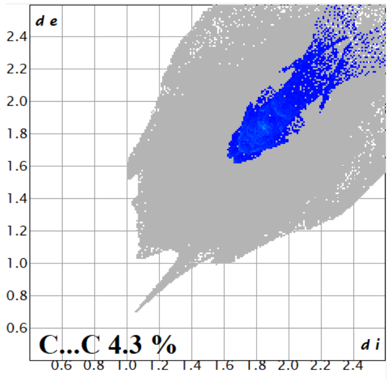

(C) **(D)**

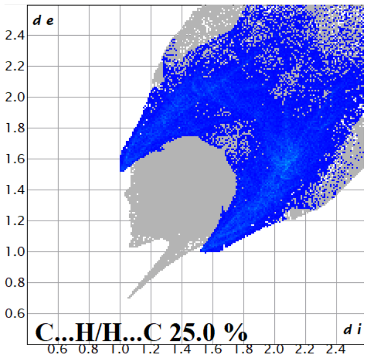

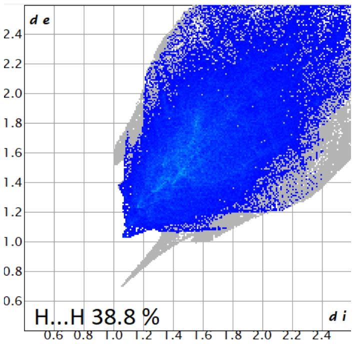

(E) **(F)**

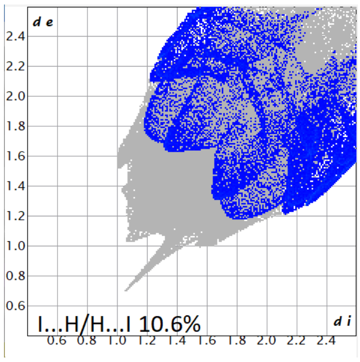

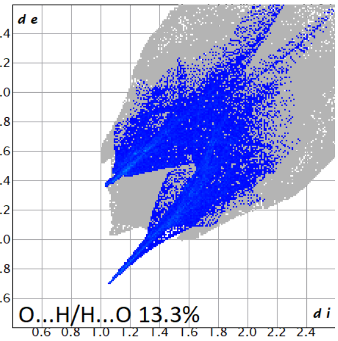

(G) **(H)**

Figure S36 Hirshfeld surface mapped oved d_{norm} (A) and shape index (B) for complex [Ru(bipy)$_2$(5-I -Sal)]·4H_2O (**8**·4H_2O) together with corresponding overall fingerprint plot (C) and plots by close contacts types: C…C (D); C…H/H…C (E); H…H (F); I…H/H…I (G) and O…H/H…O (H).

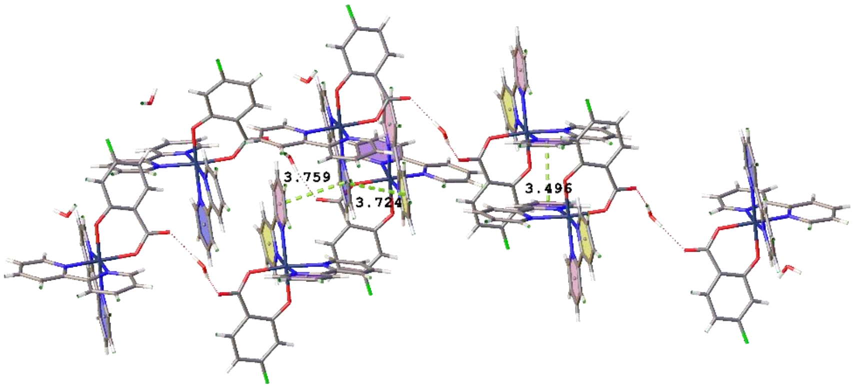

(A)

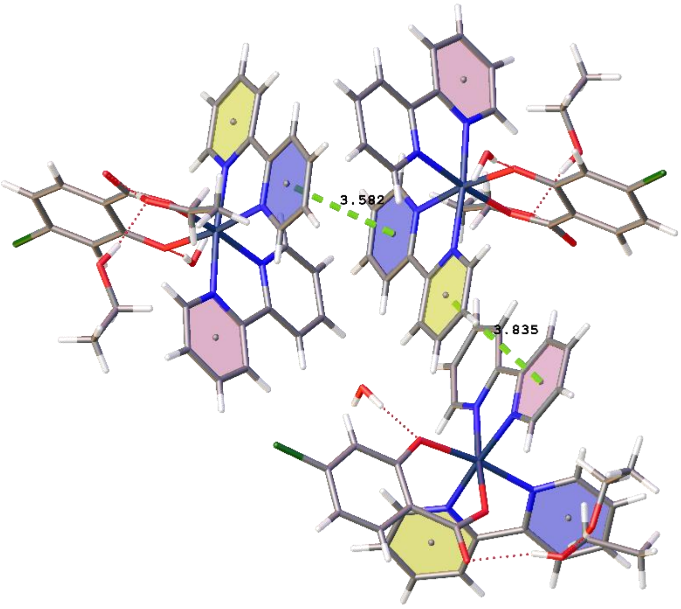

(B)

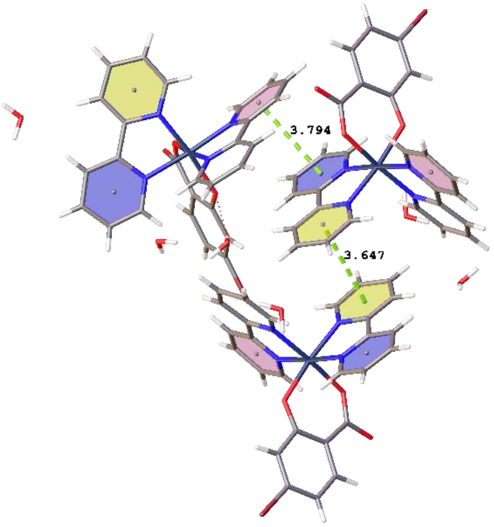

(C)

Figure S37 π···π stacking interactions for complex **1**·3H_2O·EtOH (A), **2**·2.6H_2O·2EtOH (B) and **3**·6H_2O (C).

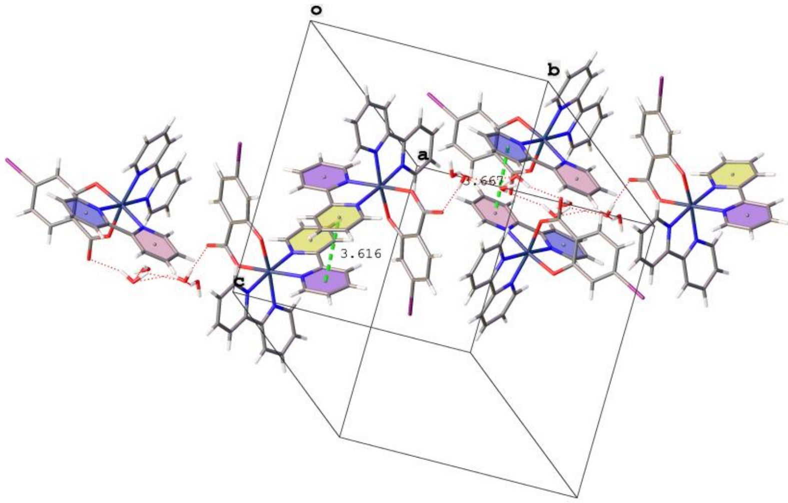

(A)

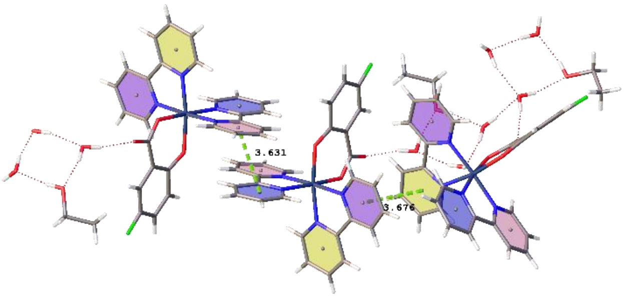

(B)

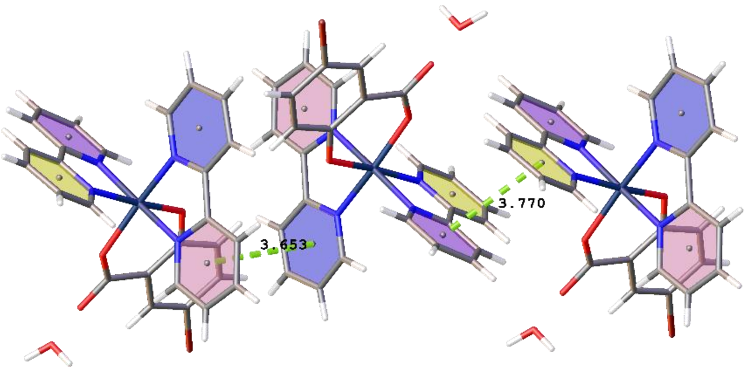

(C)

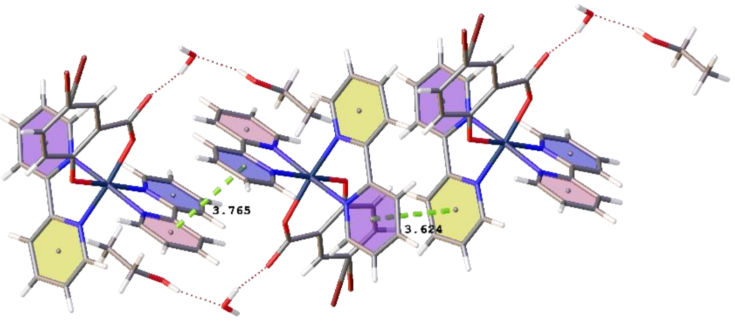

(D)

Figure S38 π···π stacking interactions for complex **4**·3H_2O (A), **5**·1.55H_2O (B), **7**·1.75H_2O (C) and **7**·4H_2O (D).

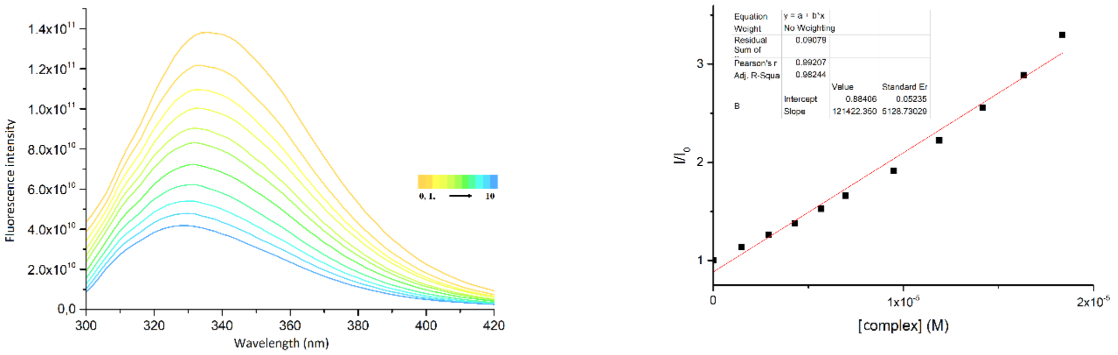

Figure S39 (A) Changes in fluorescence spectra of BSA upon complex **1** concentration rising, (B) graphical dependence of relative BSA fluorescence emission intensity (I/I_0) vs. concentration ratio [complex] .

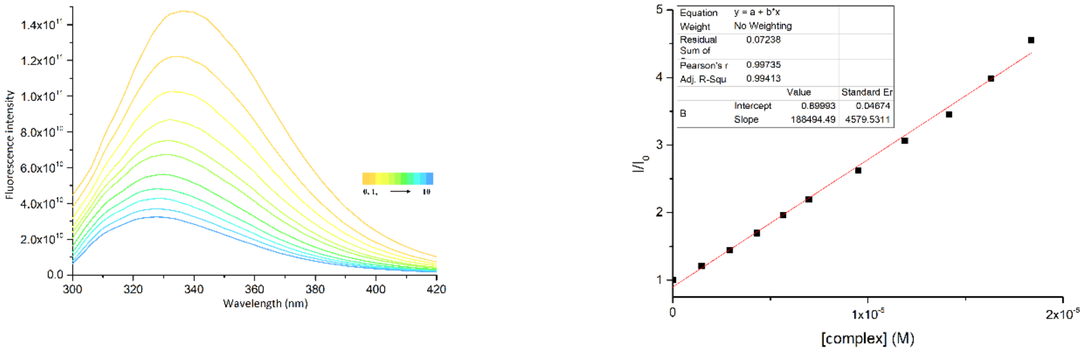

Figure S40 (A) Changes in fluorescence spectra of BSA upon complex **2** concentration rising, (B) graphical dependence of relative BSA fluorescence emission intensity (I/I_0) vs. concentration ratio [complex] .

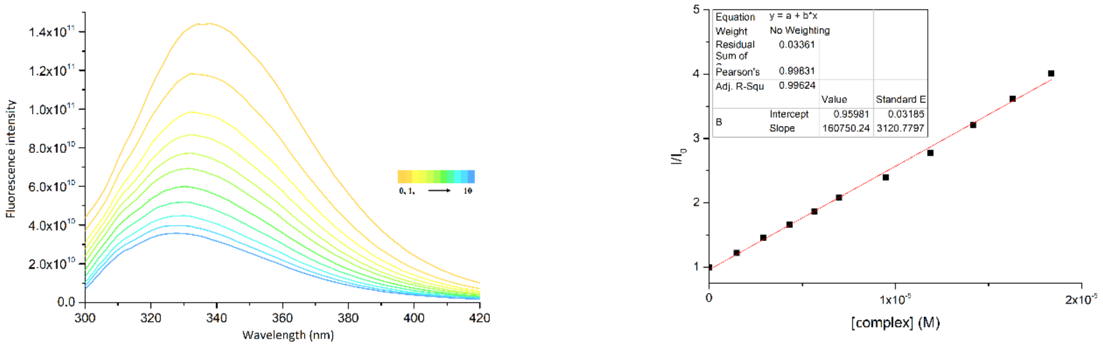

Figure S41 (A) Changes in fluorescence spectra of BSA upon complex **3** concentration rising, (B) graphical dependence of relative BSA fluorescence emission intensity (I/I_0) vs. concentration ratio [complex] .

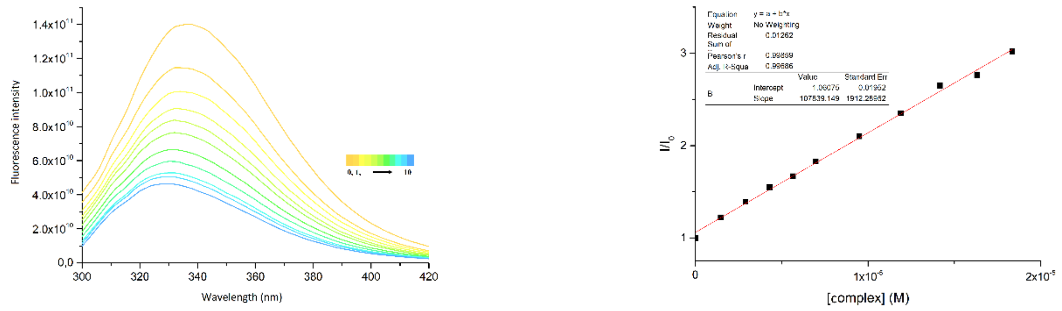

Figure S42 (A) Changes in fluorescence spectra of BSA upon complex **4** concentration rising, (B) graphical dependence of relative BSA fluorescence emission intensity (I/I_0) vs. concentration ratio [complex] .

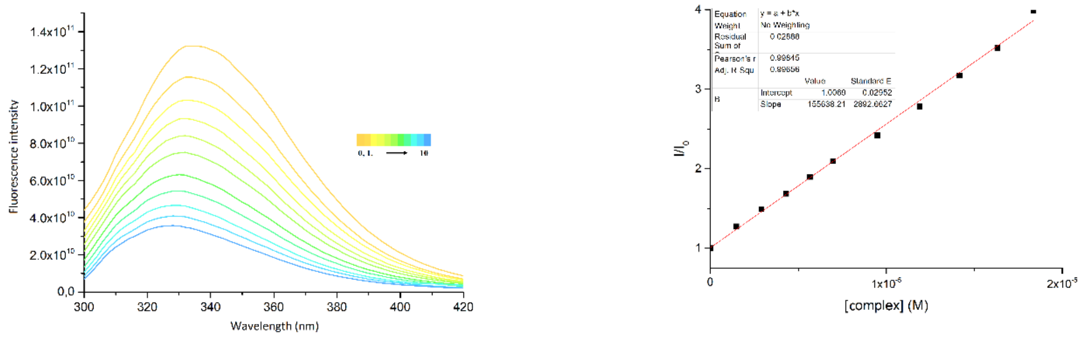

Figure S43 (A) Changes in fluorescence spectra of BSA upon complex **5** concentration rising, (B) graphical dependence of relative BSA fluorescence emission intensity (I/I_0) vs. concentration ratio [complex] .

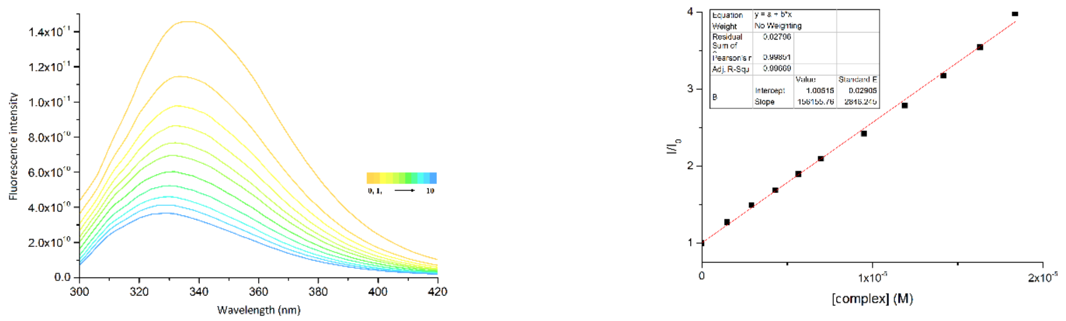

Figure S44 (A) Changes in fluorescence spectra of BSA upon complex **6** concentration rising, (B) graphical dependence of relative BSA fluorescence emission intensity (I/I_0) vs. concentration ratio [complex] .

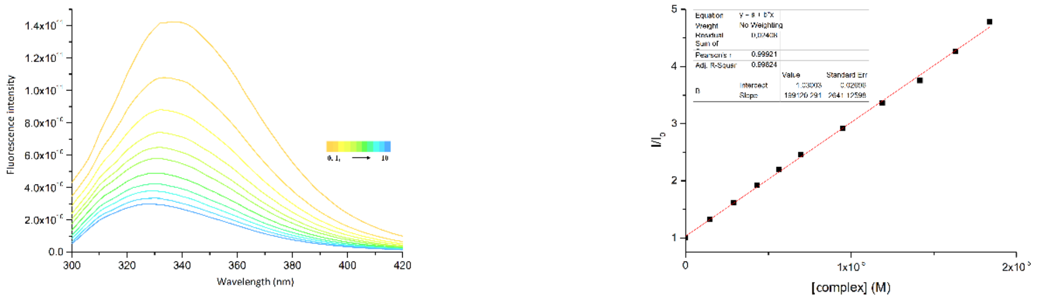

Figure S45 (A) Changes in fluorescence spectra of BSA upon complex **7** concentration rising, (B) graphical dependence of relative BSA fluorescence emission intensity (I/I_0) vs. concentration ratio [complex] .

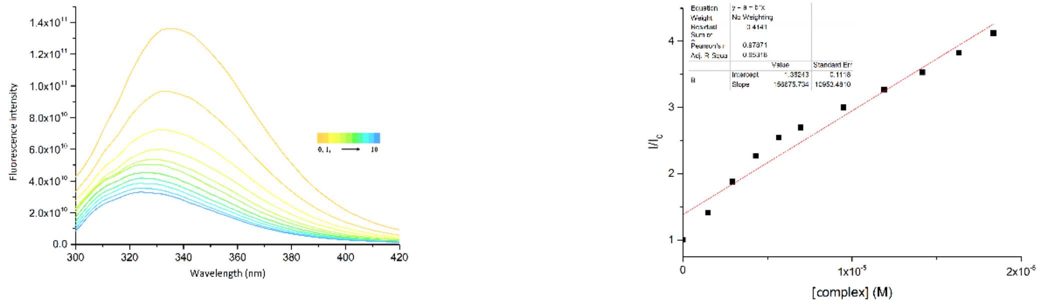

Figure S46 (A) Changes in fluorescence spectra of BSA upon complex **8** concentration rising, (B) graphical dependence of relative BSA fluorescence emission intensity (I/I_0) vs. concentration ratio [complex] .

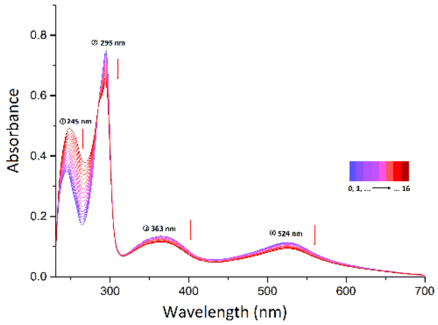

Absorbance
Wavelength (nm)
245 nm
295 nm
363 nm
524 nm
0, 1, ... → ... 16

(A)

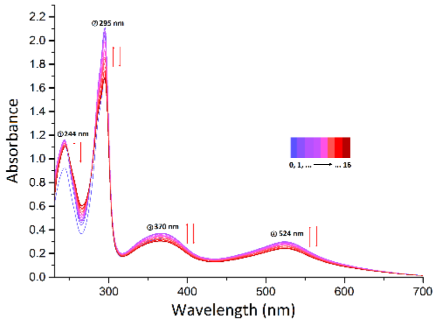

Absorbance
Wavelength (nm)
244 nm
295 nm
370 nm
524 nm
0, 1, ... → ... 16

(B)

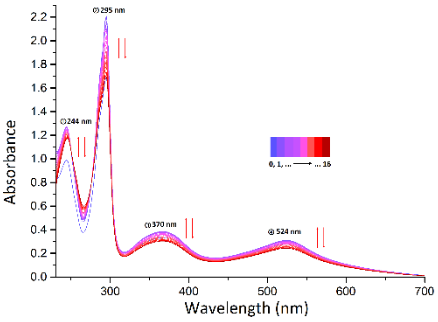

Absorbance
Wavelength (nm)
244 nm
295 nm
370 nm
524 nm
0, 1, ... → ... 16

(C)

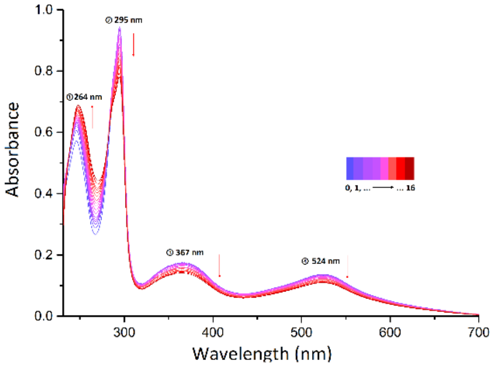

Absorbance
Wavelength (nm)
264 nm
295 nm
367 nm
524 nm
0, 1, ... → ... 16

(D)

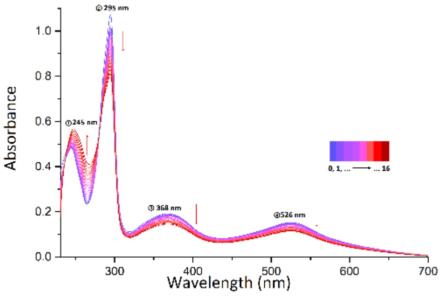

Absorbance
Wavelength (nm)
245 nm
295 nm
368 nm
526 nm
0, 1, ... → ... 16

(E)

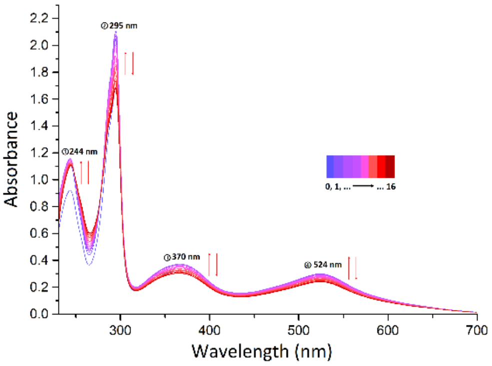

Absorbance
Wavelength (nm)
244 nm
295 nm
370 nm
524 nm
0, 1, ... → ... 16

(F)

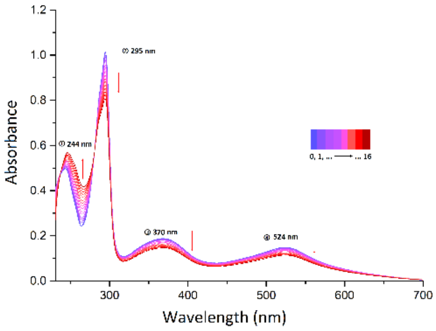

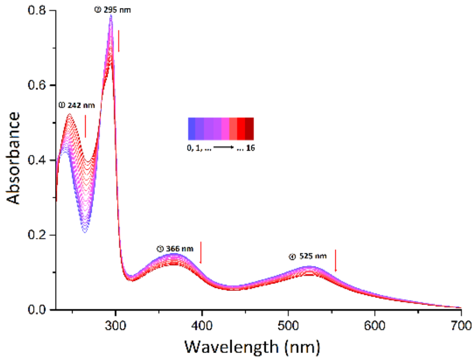

(G) **(H)**

Figure S47 Changes in the electron spectra of complexes upon addition of ct-DNA solution for complex **1** (A), **2** (B), **3** (C), **4** (D), **5** (E), **6** (F), **7** (G), **8** (H).

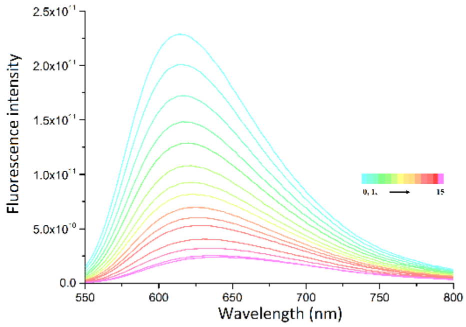

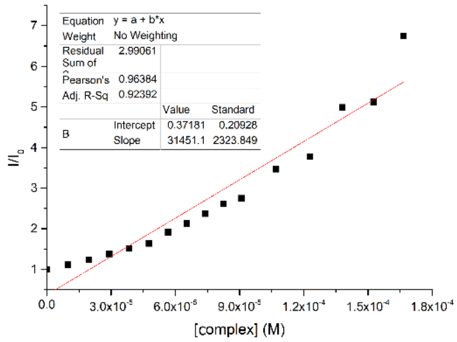

(A) **(B)**

Figure S48 (A) Changes in fluorescence spectra of EB-DNA upon complex **1** concentration rising, (B) graphical dependence of relative EB-DNA fluorescence emission intensity (*I*/*I*0) vs. concentration ratio [complex] .

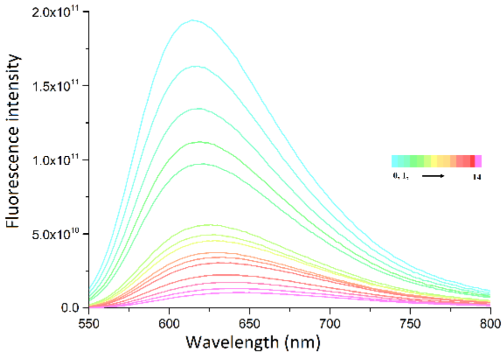

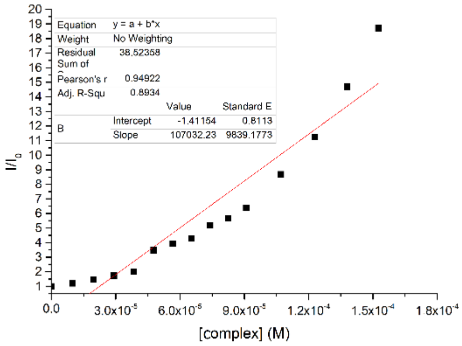

(A) **(B)**

Figure S49 (A) Changes in fluorescence spectra of EB-DNA upon complex **2** concentration rising, (B) graphical dependence of relative EB-DNA fluorescence emission intensity (*I*/*I*0) vs. concentration ratio [complex] .

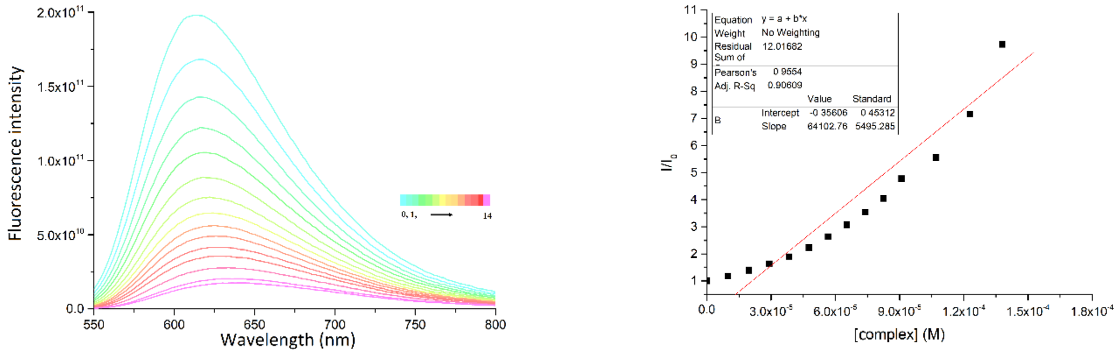

(A) **(B)**

Figure S50 (A) Changes in fluorescence spectra of EB-DNA upon complex **4** concentration rising, (B) graphical dependence of relative EB-DNA fluorescence emission intensity (*I*/*I*0) vs. concentration ratio [complex] .

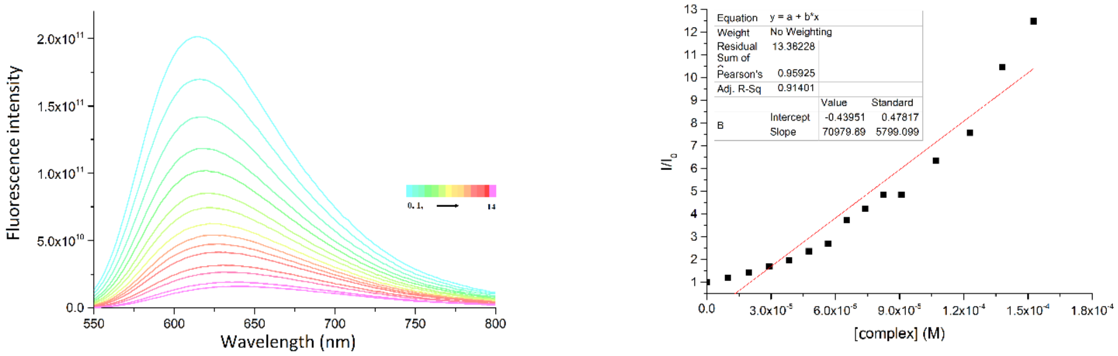

(A) **(B)**

Figure S51 (A) Changes in fluorescence spectra of EB-DNA upon complex **5** concentration rising, (B) graphical dependence of relative EB-DNA fluorescence emission intensity (*I*/*I*0) vs. concentration ratio [complex] .

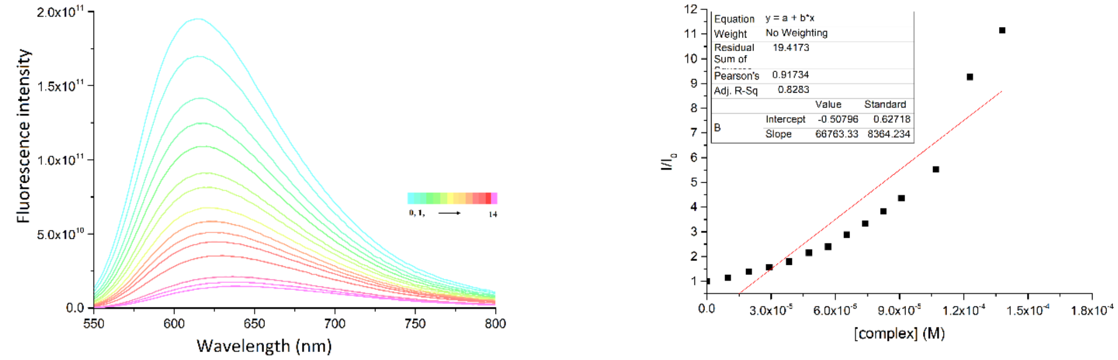

(A) **(B)**

Figure S52 (A) Changes in fluorescence spectra of EB-DNA upon complex **6** concentration rising, (B) graphical dependence of relative EB-DNA fluorescence emission intensity (*I*/*I*0) vs. concentration ratio [complex] .

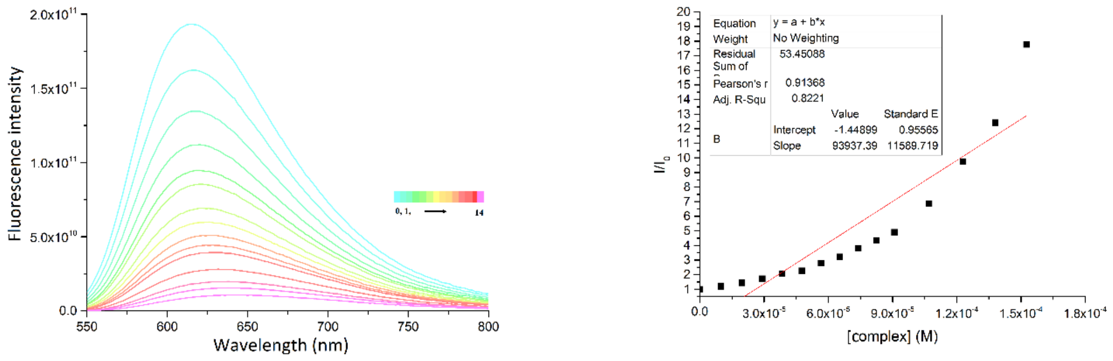

(A) **(B)**

Figure S53 (A) Changes in fluorescence spectra of EB-DNA upon complex **7** concentration rising, (B) graphical dependence of relative EB-DNA fluorescence emission intensity (I/I_0) vs. concentration ratio [complex] .

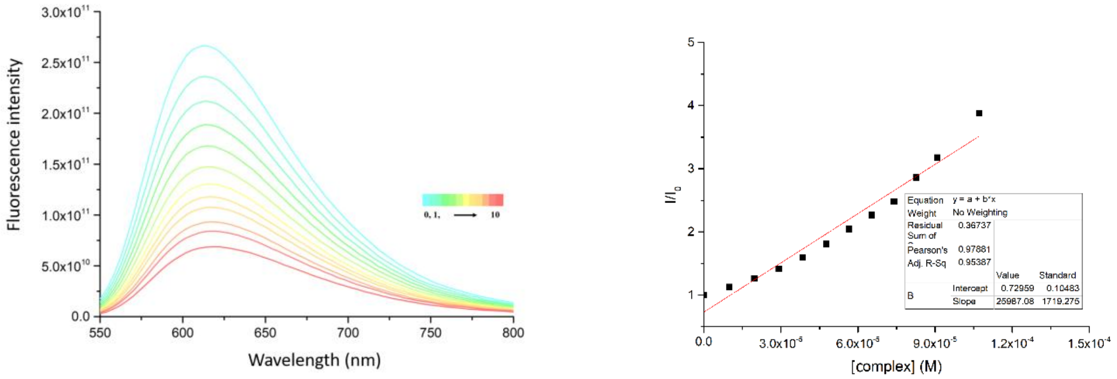

(A) **(B)**

Figure S54 (A) Changes in fluorescence spectra of EB-DNA upon complex **8** concentration rising, (B) graphical dependence of relative EB-DNA fluorescence emission intensity (I/I_0) vs. concentration ratio [complex].
